# Screening of Protein Carbonylation Sites in Human Serum by Ion Mobility Mass Spectrometry

**DOI:** 10.1021/acs.jproteome.5c00093

**Published:** 2025-06-14

**Authors:** Juan C. Rojas Echeverri, Sanja Milkovska-Stamenova, Ulf Wagner, Ralf Hoffmann

**Affiliations:** † Institute of Bioanalytical Chemistry, Faculty of Chemistry, Universität Leipzig, Leipzig 04103, Germany; ‡ Center for Biotechnology and Biomedicine, Universität Leipzig, Leipzig 04103, Germany; § Division of Rheumatology, Department of Endocrinology, Nephrology, Rheumatology, Universität Leipzig, Leipzig 04103, Germany

**Keywords:** carbonylation, aldehyde reactive probe (ARP), ion mobility spectrometry, DDA, DIA, human serum

## Abstract

Excessive oxidative stress, associated with various diseases, can induce protein carbonylation-nonenzymatic modifications involving aldehyde or keto group formation. These modifications are structurally diverse and low in abundance, which complicates their detection and quantitation. Here, we developed a strategy to identify and quantify protein carbonylation in human serum proteins from 39 rheumatoid arthritis patients and 29 healthy donors. Reactive carbonyl groups were derivatized with an aldehyde reactive probe (ARP), digested with trypsin, enriched via avidin affinity chromatography, and analyzed using RP-HPLC-ESI-IMS-MS/MS. Ion mobility spectrometry (IMS) was applied in both data-dependent (DDA) and data-independent acquisition (DIA) modes. DDA generated spectral libraries of ARP-derivatized peptides (ARP-peptides), which enabled peptide-centric detection in DIA data. We manually confirmed 86 ARP-peptides, with 93.8% of peak areas showing signal-to-background ratios >3. Among the 32 unique carbonylation sites, 28 were on human serum albumin, with hotspots at Cys58, Lys214, Lys219, Lys223, Lys456, Lys543, Lys549, and Lys565. Six previously unreported species were identified using IMS, DIA, ARP-reporter ions, and *de novo* sequencing. The ARP-peptides were quantified with ≥ 75% intrabatch reproducibility (coefficient of variation <20%). Similar modification levels were observed in both groups, suggesting basal, disease-independent carbonylation in abundant serum proteins.

## Graphical abstract

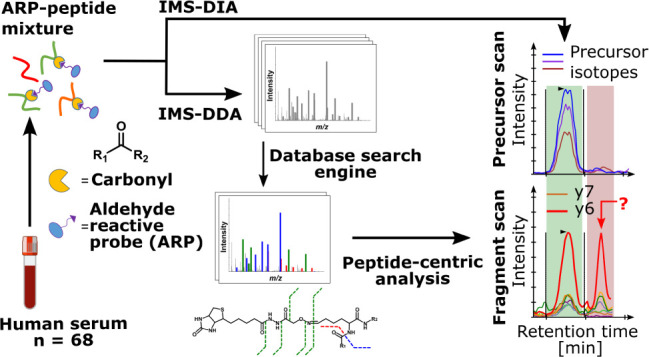

## Introduction

Protein carbonylation refers to nonenzymatic post-translational modifications (PTMs) that yield “reactive carbonyls”, i.e., aldehydes and ketones, which are considered a hallmark of oxidative damage.^1^ They are formed at higher levels under conditions of excessive oxidative stress^1,2^ by (i) direct amino acid oxidation through metal-catalyzed reactions,^3^ (ii) adduct formation with highly reactive (di)­carbonyl compounds, such as reactions between reducing sugars and proteins including further oxidation products of these sugar adducts (advanced glycation end products, AGEs),^4^ and (iii) reaction with advanced lipoxidation end products (ALEs).^5^ These modifications tend to accumulate in electron-rich residues.^1,6,7^ Carbonyls generated by metal-catalyzed oxidation (MCO) *in vivo* are typically restricted to the vicinity of coordinating metal-binding sites due to the short lifetime of the hydroxyl radical that initiates these reactions. Reactive carbonyl species (RCS), being electrophilic, preferentially form adducts with nucleophilic amino acids such as lysine, cysteine, arginine, and histidine. As carbonylation is irreversible in cells or body fluids, the products can accumulate and trigger further reactions. These include carbonyl-induced protein cross-linking, which can alter protein function and reduce proteolytic degradation, resulting in the accumulation of protein aggregates.^8^ Protein carbonylation has been described in the context of several diseases characterized by systemic chronic inflammation (SCI), including Alzheimer’s disease (AD), diabetes, and rheumatoid arthritis (RA).^9–11^ Currently, the most important laboratory parameters in the management of rheumatoid arthritis (RA) include serologic testing for rheumatoid factor (RF), anticitrullinated protein antibodies (ACPA), and inflammatory markers such as C-reactive protein (CRP).^12^ However, RF positivity and ACPA specificity identify only 60–70% and 60–75% of RA patients, respectively, suggesting the need for additional disease-specific markers that could be found in carbonylated proteins.^13^

There is some evidence linking carbonylation levels to inflammatory diseases, but the identification and quantification of carbonylation sites remain challenging due to the low stoichiometric abundance of these PTMs in the proteome. However, accurate analysis of these modifications is a prerequisite for distinguishing between states of physiological oxidative stress (eustress) and deleterious oxidative stress (distress), which is essential for better characterization of disease pathogenesis and possible translation into a clinical setting.^14^ The use of carbonyl-specific derivatization tags for enrichment has improved the analysis of carbonylated proteins and the mapping of carbonylation sites in biological samples.^1,7^ These tags are typically based on hydrazine- or hydroxylamine-based reagents with a biotin moiety that allows their enrichment by avidin affinity chromatography at the protein or peptide level.^15–17^ Hydroxylamine-based reactive probes, such as *N*’-aminooxymethylcarbonylhydrazino-d-biotin (a.k.a. aldehyde reactive probe, ARP), are preferred for derivatization due to the formation of stable oximes without the need for subsequent reduction.^17^ They also provide higher yields in acidic conditions for some reactive carbonyls.^18^ Affinity enrichment at the peptide level improves the identification of carbonylation sites by reducing the background of nonderivatized peptides, allowing mass spectrometry-based approaches to better profile analytes of interest.

In this study, reactive carbonyl groups in proteins were specifically derivatized and, after tryptic digestion, the derivatized peptides were enriched by affinity chromatography and analyzed by reversed-phase high-performance liquid chromatography (RP-HPLC) coupled online to electrospray ionization and mass spectrometry (MS) detection. The identification and quantitation of carbonylation sites were enhanced by 1) integrated ion mobility spectrometry (IMS) to improve peak capacity and sensitivity,^19^ 2) data-independent acquisition (DIA) to target low-abundance compounds,^20^ and 3) data validation in Skyline^21–23^ with a peptide-centric approach that incorporated accurate mass, ion mobility, retention time information, and peptide spectral matching (PSM) to improve the identification of weak signals. These strategies have been successfully applied to the study of various PTMs,^24,25^ but they have not been applied to derivatized carbonylated proteins. We relied on our previously reported bottom-up proteomics workflow enriching ARP-labeled carbonylated peptides (ARP-peptides).^20^ A spectral library generated from data sets collected in data-dependent acquisition (DDA) modes was used to analyze serum samples collected from 39 patients diagnosed with rheumatoid arthritis and 29 samples from healthy individuals using the DIA mode. A total of 86 carbonylated peptides from 11 protein groups were present in all 68 serum samples. Although no differences were found in the occurrence and abundance of these species between RA patients and healthy controls, we were able to quantify carbonylation sites in a complex biofluid that were previously only determined *in vitro*.

## Experimental Procedures

### Materials

Materials were obtained from the following suppliers: AppliChem GmbH (Darmstadt, Germany): iodoacetamide (IAA, ≥99%) and tris­(hydroxymethyl)­aminomethane (Tris, ≥99.9%); Biosolve GmbH (Valkenswaard, The Netherlands): acetonitrile (ultrahigh-performance liquid chromatography–mass spectrometry (ULC-MS) grade, ≥99.97%), formic acid (ULC-MS grade, ≥99%), and methanol (ULC-MS-grade, ≥99.98%); Carl Roth GmbH (Karlsruhe, Germany): dithiothreitol (DTT, ≥99%), hydrogen peroxide (30%), and urea (≥99.5% p.a.); Cayman Chemical (Ann Arbor, USA): Aldehyde Reactive Probe (ARP; trifluoroacetate salt); Greiner Bio-One GmbH (Frickenhausen, Germany): low-binding 96-well microtiter plates; Merck KGaA (Darmstadt, Germany): microcon-10 kDa molecular weight cutoff (MWCO) regenerated cellulose centrifugal filters; Pierce Biotechnology (Rockford, IL, USA): 50% slurry of immobilized monomeric avidin on agarose beads and spin columns, bovine serum albumin (BSA) standard; SERVA Electrophoresis GmbH (Heidelberg, Germany): Coomassie Brilliant Blue G 250; Sigma-Aldrich Chemie GmbH (Steinheim, Germany): ammonium bicarbonate (≥99.5%), hydrochloric acid (HCl, 36.5–38%), sodium chloride (NaCl, ≥99.5%), sodium deoxycholate (≥97%), sodium phosphate dibasic dodecahydrate (≥99.0%), sodium phosphate monobasic (≥99.0%), and TPCK-treated trypsin from bovine pancreas. Water was purified in-house (resistivity >18 MΩ·cm^–1^; total organic content <10 ppb) using a PureLab Ultra Analytic System (ELGA Lab Water, Celle, Germany).

### Study Population

Serum samples were collected from 39 patients (30 females and 9 males) meeting the ACR/EULAR 2010 classification criteria for rheumatoid arthritis^12^ recruited from the Rheumatology Clinic at Leipzig University, Germany. Age-matched serum samples were also collected from 29 healthy blood donors (controls: 16 females and 13 males). Written informed consent was obtained from all 68 donors. This study was conducted in accordance with the tenets of the Declaration of Helsinki and was approved by the Ethics Committee of Leipzig University (approval no: 430/16-ek).

### Serum Sample Preparation

Whole blood was collected in serum tubes, centrifuged at room temperature (3275 g, 10 min), and stored at −80 °C. Serum samples were thawed on ice and divided into four aliquots (100 μL) for individual analysis. Additionally, a total serum pool was prepared by mixing aliquots (50 μL) of all serum samples, dividing them into aliquots (250 μL), and storing them at −80 °C until analysis.

### Protein Quantitation

Aliquots of serum samples were thawed on ice and diluted 100-fold with ammonium bicarbonate (0.1 mol/L), and an aliquot (5 μL) was mixed with Bradford solution (0.1 g/L Coomassie Brilliant Blue G-250 in an aqueous mixture of ethanol (5%, v/v) and phosphoric acid (10%, v/v)) in 96-well microtiter plates. Absorbance was recorded at 595 nm using a SpectraMAX Paradigm microplate reader (Molecular Devices, San Jose, USA). The plate contained triplicate dilutions of a total pool reference and a 2-fold dilution series of BSA from 1 g/L to 62.5 mg/L, which was used to estimate the protein concentration of each serum sample (Table S1).

### Block Randomization and Sample Design

The RA and control samples were block-randomized into two batches, matched for clinical classification and sex (Figure S1 and Table S2). Due to instrumental limitations, the samples had to be divided and prepared in two batches, with each batch containing three serum pool samples (sample preparation quality controls, SPQCs) prepared in parallel with the donor samples. An additional serum pool sample was included, prepared by the same procedure except that ARP was not added in the derivatization step. These samples were used as matrix-matched negative controls (NCs) to verify the authenticity of the derivatized products and to facilitate the detection of false-positive identifications.

### Protein Carbonyl Derivatization and Protein Digestion

Serum samples (2 mg protein) were diluted with formic acid (1% v/v) to a final volume of 500 μL, transferred to preconditioned 10 kDa ultrafiltration units, and centrifuged (14,000 × g, 30 min, 25 °C). Formic acid (1% v/v, 500 μL) was added, and the samples were centrifuged again (14,000 × g, 30 min, 25 °C); this step was repeated twice. ARP dissolved in water (25 mmol/L, 40 μL) and formic acid (1% v/v, 200 μL) were added and incubated overnight at room temperature in the dark with gentle shaking (300 rpm). The solution was neutralized by adding a sodium hydroxide solution (40 μL, 1 mol/L). A solution of sodium deoxycholate (SDC, 0.5% w/v, 300 μL) in ammonium bicarbonate (0.1 mol/L) was added, mixed, and centrifuged (50 min, 14,000 g, 25 °C).^20^ Additional SDC solution (440 μL) was added and spiked with in-house-expressed DnaK (UniProt ID: P99110)^26^ as an internal control protein. After centrifugation (50 min, 14,000 g, 25 °C), aqueous DTT solution (12.5 μL, 500 mmol/L) was added, incubated for 1 h (37 °C, 550 rpm), and centrifuged (20 min, 14,000 g, 25 °C). SDC solution (200 μL) was added and centrifuged (20 min, 14,000 g, 25 °C). This step was repeated once before adding iodoacetamide (100 μL, 50 mmol/L). After 20 min in the dark at RT, samples were centrifuged (10 min, 14,000 g, 25 °C), and SDC solution (100 μL) was added and centrifuged (25 min, 14,000 g, 25 °C). The addition of SDC and centrifugation was repeated three more times. Trypsin (80 μg, 32 μL) dissolved in ammonium bicarbonate solution (0.1 mol/L) was added (protein-to-enzyme ratio of 25:1) and incubated overnight in a humidity chamber at 37 °C. The digest was collected by centrifugation (10 min, 14,000 g, 25 °C). Ammonium bicarbonate solution (50 μL) was added to the filters and centrifuged (15 min, 14,000 g, 25 °C). This step was repeated twice, with the last centrifugation lasting 30 min. The combined solutions were acidified with TFA (5.6 μL) to precipitate the SDC.^27^ Ethyl acetate (300 μL) was added to dissolve the SDC, vortexed (10 s), and centrifuged (2 min, 15,700 g, 25 °C) to facilitate phase separation, and the organic phase containing the SDC was discarded. This extraction was repeated twice. Samples were dried under vacuum (100 mbar for 30 min followed by 1 mbar for 3.5 h). The digests were dissolved in 1 mL aqueous acetonitrile (30% v/v) containing formic acid (0.1% v/v), and aliquots (25 μL) were stored (nonenriched fraction), while the remaining solutions were dried under vacuum and stored at −20 °C. An aliquot of the nonenriched fractions (5 μL) was taken from each sample, diluted with aqueous acetonitrile (30% v/v, 0.5 mL), and divided as follows: four aliquots (100 μL) were taken for the analysis of donor samples, while one aliquot (70 μL) was combined with the corresponding aliquots of the other samples to obtain a nonenriched fraction quality control (NEF-QC) and divided into aliquots of 250 μL (Figure S1; middle panel). All samples were stored at −20 °C.

### Biotin-Avidin Affinity Chromatography

Mini-spin columns packed with monomeric avidin agarose beads (50% slurry, 200 μL) were washed with phosphate buffer (1.5 mL, 10 mmol/L phosphate, pH 7.4) and equilibrated with phosphate-buffered saline (PBS, 2 mL, 20 mmol/L phosphate, 300 mmol/L NaCl, pH 7.4). Dried samples were reconstituted in PBS (1 mL), applied to the column, and washed with PBS (1 mL), phosphate buffer (1 mL), ammonium bicarbonate (50 mmol/L) in aqueous methanol (20% v/v, 2 mL), and water (1 mL). Elution was performed with aqueous acetonitrile (800 μL, 30% v/v) containing formic acid (0.4% v/v) at a flow rate of ∼500 μL for 17 to 20 min under gravity. The acetonitrile was evaporated under vacuum (100 mbar for 30 min and 1 mbar for 1.5 h), and the remaining solution was transferred to preconditioned 10 kDa ultrafiltration units. Aqueous acetonitrile (1% v/v, 500 μL) containing formic acid (0.1% v/v) was added and centrifuged (25 min, 14,000 g, 25 °C) to trapun bound monomeric avidin in the filters. The tubes used to collect the elution fractions from the mini-spin columns were washed once with 50 μL of aqueous acetonitrile (1% v/v) containing formic acid (0.1% v/v), vortexed, and briefly spun, and the solution was also transferred to the respective ultrafiltration units and centrifuged again (25 min, 14,000 g, 25 °C). The filtrates were dried under vacuum (1 mbar, 4 h) and dissolved in 100 μL of aqueous acetonitrile (3% v/v) containing formic acid (0.1% v/v). This solution was divided into three groups (Figure S1; middle panel): (i) three aliquots (17 μL) as individual enriched samples, (ii) one aliquot (17 μL) to prepare an RA and one control-enriched digest pool, and (iii) one aliquot (15 μL) to be combined with similar aliquots of the other samples (except ARP-negative controls) and divided into aliquots (50 μL) as enriched fraction quality control (EF-QC) samples. All samples were stored at −20 °C.

### Mass Spectrometry Data Acquisition

First, the ideal sample amount loaded onto the column to obtain the highest signal intensities and reproducible separations without carryover was determined using NEF-QCs (25 ng of original protein, 0.0013% of sample) for the nonenriched fractions and EF-QCs (5.0% of the reconstituted enriched fractions, 4.9% of sample) for the enriched fractions. Peptides were separated on a nanoACQUITY Ultra Performance LC (Waters Corp., Manchester, UK) coupled online to a Q-TOF SYNAPT G2-S*i* instrument (Waters) using optimal sample quantities. Peptides were trapped on a nanoACQUITY Symmetry C_18_-column (internal diameter (ID) 180 μm, length 2 cm, particle diameter 5 μm) at a flow rate of 5 μL/min using 1% (v/v) aqueous acetonitrile containing 0.1% (v/v) formic acid for 6 min. Separation was performed on a BEH 130 column (C_18_-phase, ID 75 μm, length 10 cm, particle diameter 1.7 μm; 35 °C) using water and acetonitrile containing formic acid (0.1% (v/v)) as eluents A and B, respectively. After trapping, peptides were separated using linear gradients of 3% to 33.8% B (61.5 min, 0.3 μL/min), held isocratically for 0.5 min, 33.8% to 40% B (12 min, 0.4 μL/min), and 40% to 95% B (10 min, 0.4 μL/min). After 2 min, the content of eluent B was reduced from 90% to 1% in 5 min (0.3 μL/min), and the column was equilibrated for 10 min. The nanospray source used a PicoTip emitter (New Objective, Littleton, US) with a spray voltage of 3 kV, a sampling cone of 30 V, a source offset of 80 V, a source temperature of 100 °C, a cone gas flow of 20 L/h, and a nanoflow gas pressure of 0.2 bar. Argon (99.998%) was used as the collision gas and was introduced at a flow rate of 2 mL/min. For traveling wave IMS (TWIMS), helium (99.999%) was used for the pre-TWIMS cell and nitrogen (99.999%) for the TWIMS cell at flow rates of 180 and 90 mL/min, respectively. For all MS acquisition modes used, data were collected in positive ion mode, and reference scans of Glu-fibrinopeptide B were acquired every 30 s for postacquisition lock mass recalibration using the doubly protonated ion at *m*/*z* 785.842.

Two IMS-based DDA experimental setups, HD-DDA Trap and HD-DDA Transfer, were adapted from previously reported methods (Supporting Information for details)^23^ to analyze NEF-QC and EF-QC samples, as well as RA- and control-specific enriched digest pools, to generate spectral libraries and refine retention time indexing of modified peptides (Figure S1; lower panel). Analysis of RA and control pools was performed 6 weeks after data collection of individual donor samples using precursor ion preference lists. These cohort-specific pools were analyzed to capture modified peptides that might be overrepresented in only one cohort. The initial precursor ion targets in the preference lists were generated based on previously identified ARP-peptides^20^ and preliminary analysis of pooled serum samples. They were updated as new DDA data were acquired. Retention times were calibrated for each acquisition queue, and product ion spectra of precursor ions in the preference lists were acquired for up to 2.0 s. Of note, when using preference lists on Synapt G2-S*i* instruments, targets are prioritized for fragmentation, but other ions were still considered for fragmentation.

Data intended for relative quantitation evaluations were acquired with wideband DIA using drift-time-specific collision energies.^28^ In this UDMS^E^ mode, low-energy (LE) scans (*m*/*z* range 50–2000) and high-energy (HE) scans (*m*/*z* range 50–2000) were acquired in resolution mode (*R* = 20,000 at *m*/*z* 400; fwhm) using MS scan times of 0.4 and 0.8 s for LE and HE scans, respectively. IMS consisted of full TWIMS cycles with a ramped wave velocity of 500 to 1200 m/s and a wave height of 40 V, pre-IMS trapping for 500 μs at 15 V, 0 V extraction, and an IMS delay of 1 ms after trap release. Precursor ions were transferred through the post-TWIMS transfer cell with an acceleration energy of 2.0 eV during the LE scan and drift-time-specific collision energies for HE scans: bins 1–19:4.0 eV, bins 20–120:16.3–60.7 eV, bins 121–195:60.7–65.7 eV, and bins 196–200:4.0 eV. Collision energies were optimized according to Water’s standard UDMS^E^ optimization procedure using a tryptic digest of cytosolic proteins.

The control and RA sample acquisition queues were measured continuously and were accompanied by fraction-specific QC samples. For the enriched fractions, EF-QC samples were measured with HD-DDA (with and without preference lists) and UDMS^E^, while all donor samples were measured with UDMS^E^ in the following order (Figure S1; bottom panel): (i) initial HD-DDA measurements (with and without preference lists) to condition the system and extract retention times to update precursor ion targeting in the scheduled preference list, (ii) triplicate instrumental replicates of EF-QCs with UDMS^E^ to evaluate system performance, (iii) Batch 1 SPQC samples, (iv) Batch 2 SPQC samples, (v) UDMS^E^ measurements of the EF-QC sample followed by ten Batch 1 donor samples and an EF-QC repeated for all Batch 1 samples, (vi) EF-QCs with HD-DDA with scheduled preference lists, (vii) UDMS^E^ measurement of the EF-QC followed by ten Batch 2 donor samples and an EF-QC using UDMS^E^ repeated for all Batch 2 samples, and (viii) NC and blank samples.

### Peptide Identification

HD-DDA Transfer and HD-DDA Trap files were processed separately to obtain method-specific spectral libraries for the nonenriched and enriched fractions. The DDA LC-IMS-MS/MS raw files were imported into PEAKS Studio v10.5 (Bioinformatics Solutions, Waterloo, Canada), and a lock-mass correction was applied using the PEAKS built-in loader with the double protonated signal of Glu-fibrinopeptide B at *m*/*z* 785.842, considering a detection error tolerance of 0.5 Da. Tandem mass spectra were processed using *de novo* sequencing with cysteine carbamidomethylation (+57.022 Da) and methionine oxidation (+15.995 Da) as variable modifications and searched against the human SwissProt protein database (accessed on 2024-05-17), the chaperone protein sequence of DnaK (UniProt ID: P99110), and the common repository of adventitious proteins (cRAP) contaminant database (https://www.thegpm.org/crap/; accessed on 2024-05-17), requiring tryptic cleavage of at least one of the peptide termini. Proteins were first identified using the modifications mentioned for *de novo* sequencing and database matching (first pass) to obtain a list of identified proteins, which was used to identify modified proteins with PEAKS PTM (second pass), considering in-built 312 post-translational modifications (PTMs) reported in UNIMOD. Searches of the enriched fractions additionally included a customized list of ARP-labeled modifications (Table S3). Methionine oxidation and cysteine carbamidomethylation were always considered as variable modifications. The database search initially allowed for up to three missed cleavage sites, a precursor ion tolerance of 30 ppm, and a fragment ion tolerance of 25 mDa. After reviewing the tandem mass spectra of the ARP-modified peptides, stricter PTM search settings were applied: 1 missed cleavage site, precursor ion tolerance reduced to 15 ppm, and only UNIMOD PTMs with more than 150 PSMs were included. These results were used to generate the DDA spectral libraries for downstream analysis. All peptide identification results were filtered with a 1% false discovery rate (FDR) at the peptide level. The peptide spectrum match (PSM) identification results were exported as text tables and a *pepXML* summary. The raw files were converted using PEAKS Studio version 10.5 and exported as *mzXML* files.

### Data Analysis in Skyline

For each of the implemented HD-DDA methods (i.e., HD-DDA Transfer and HD-DDA Trap), separate spectral libraries were generated with Skyline-daily v23.1.1.459 for the nonenriched and enriched fractions using the .*pepXML* and .*mzXML* files from PEAKS Studio. The FDR filtration was based on PEAKS Studio; i.e., no additional PSM filtration was applied to create the spectral libraries. The data corresponding to each of the fractions were processed separately with more critical manual validation required for the enriched fractions, focusing on the analysis of modified peptides. DDA and DIA data were analyzed in parallel using Skyline, as reported recently (Supporting Information).^23^ DDA files were used to validate confident ARP-labeled peptides for the presence of ARP reporter ions^29^ and sufficient b- and y-ion sequence coverage to allow accurate PTM mass shift localization. ARP-labeled peptides with matching extracted ion chromatograms (XICs) in the NC samples were considered false positives and were removed from the list. Besides being used as an additional filtration criterion, NCs were also used to estimate the average background signal generated by nonderivatized peptides that remain in enriched fractions due to unspecific interactions. However, NCs were not used to normalize ARP-labeled peptide peak areas used for quantitative comparisons. The validated list was used to identify the ARP-labeled peptides in UDMS^E^ data sets acquired from EF-QC samples. This list was used to create an ion mobility library and an indexed retention time calculator using endogenous peptides as the indexed retention time (iRT) standards. Both were then used to import the results of the donor samples, followed by the final curation of the chromatographic peaks. The mass spectrometry data and refined Skyline documents are provided at https://panoramaweb.org/HumanSerumCarbonylationRA.url, and the assigned ProteomeXchange identifier is PXD058666. The confirmed modified peptides and precursor ion integrated areas from each donor from both nonenriched and enriched fractions were exported as custom reports for subsequent analysis in R.

### Data Visualization and Statistical Analysis

Data generated by Skyline, PEAKS Studio, and mgfHunter were integrated into an R notebook (available at https://panoramaweb.org/HumanSerumCarbonylationRA.url) for processing and visualization. Differential analysis was performed using MSstatsPTM^30^ with the following considerations for normalization: (i) integrated areas of modified peptides were normalized with respect to the integrated area of their respective proteins using the top 5 (intensity-wise) peptides of these proteins measured in the nonenriched fractions and (ii) normalization at the protein level was chosen instead of normalization of unmodified peptides because trypsin cleavage is reduced or prevented at modified lysine residues. The statistical analysis was divided into two parts: one considering both batches of samples to account for batch effects and a second, more conservative analysis considering only the samples from the first batch.

## Results

### ARP-Peptide Spectral Libraries

The preferred quantitation-focused DIA approach required a valid spectral library, which was obtained using the IMS capability of the Synapt G2-S*i* by analyzing the pooled samples in the HD-DDA Trap mode with wideband enhancement^31^ to obtain intense fragment ion signals, including the ARP-specific reporter ions. This wideband enhancement mode tends to miss multiply charged fragment ions and thus does not provide good sequence coverage for multiply protonated peptides. Higher charged precursor ions that provided insufficient sequence coverage but displayed ARP-specific reporter ions were used to generate an inclusion list to be targeted in a second analysis using the HD-DDA Transfer method. This method provides better sequence coverage due to the presence of singly and multiply charged fragment ions. Combining the output of both methods resulted in the identification of 899 ARP-derivatized peptides (Figure S2 and Table S4). However, 324 (36.0%) identified peptides were not derivatized at a carbonyl group but originated from reactions with Asn/Gln deamidation intermediates and Asp/Glu isomerization intermediates.^20^ The remaining sequences were manually verified by the ARP-reporter and backbone fragmentation signals, finally resulting in a total of 86 presumably ARP-derivatized carbonylated peptides (Table S5) with the carbonylation site uniquely identified by the increment mass observed in the b- or y-ion series and detected in all QC samples with consistent indexed retention time (iRT), precursor ion mobility, and precursor isotope ratios. Although various modification-specific ARP fragmentation patterns have been observed,^20,32^ we used only the most commonly reported fragmentation patterns that generate relatively intense reporter ion signals at *m*/*z* 332.14, 299.12, 259.12, and 227.08. While all of the peptides displayed intense reporter ions, the signal ratios varied. The signal at *m*/*z* 227.08, corresponding to the biotin moiety of ARP, was consistently the most intense reporter ion signal, as shown for peptide LKCASLQK (residues 222 to 229 of human serum albumin (HSA)), which was observed with different modifications at position 2 of the sequence, identified by the signals of the b_2_- and y_7_-ions (Figure 1). These 86 peptides represented 50 modification sites in 15 proteins belonging to 11 protein groups (Table 1), of which 72 (87%) corresponded to 32 modification sites in HSA, including Cys58, Lys214, Lys219, Lys223, Lys456, Lys543, Lys549, and Lys565 as hotspots carrying multiple modification types (Table 2).

**Table 1:** Carbonylation Sites Identified in Proteins and the Mass Shifts Observed upon ARP-Derivatization with Carbonylation Sites^a^

Protein accession	Protein	ARP-related mass shift	Carbonylation sites
P02768	Human serum albumin	+270.067, +281.112, +298.110, +311.105, +312.089, +329.115, +355.131, +367.131, +369.147, +371.126, +381.147, +383.138, +385.142, +395.138, +399.121, +409.142, +419.126, +457.163, +475.174	K28, K36, Q57, C58, P59, T103, T107, M111, K130, N135, P137, K160, K186, Q194, R210, K214, K219, K223, K249, K257, K300, M322, K375, K456, K460, K463, T520, K543, K549, K565, M572, K588
P01857*	Immunoglobulin heavy constant gamma 1	+281.1124, +329.1158	M135, H151
P01859*	Immunoglobulin heavy constant gamma 2		M131, H147
P01860*	Immunoglobulin heavy constant gamma 3		M182, H198
P02647	Apolipoprotein A-I	+281.1124	M136, M172
B9A064	Immunoglobulin lambda-like polypeptide 5	+312.089	K158
P01861	Immunoglobulin heavy constant gamma 4	+281.1124	M132
P02652	Apolipoprotein A-II	+312.0892	K69
P02787	Serotransferrin	+367.131	C260
P04196	Histidine-rich glycoprotein	+312.089	K445
P0CG04	Immunoglobulin lambda constant 1	+312.089	K50
P0DOY2^#^	Immunoglobulin lambda constant 2	+312.089	K50
P0DOY3^#^	Immunoglobulin lambda constant 3		K50
Q14624	Interalpha-trypsin inhibitor heavy chain H4	+312.089	K113
Q66K66	Transmembrane protein 198	+311.105	T7

a $ Modification site is based on the canonical protein sequence deposited in www.uniprot.org.* and # denote immunoglobulins identified by the same peptide sequence.

**Figure 1:**
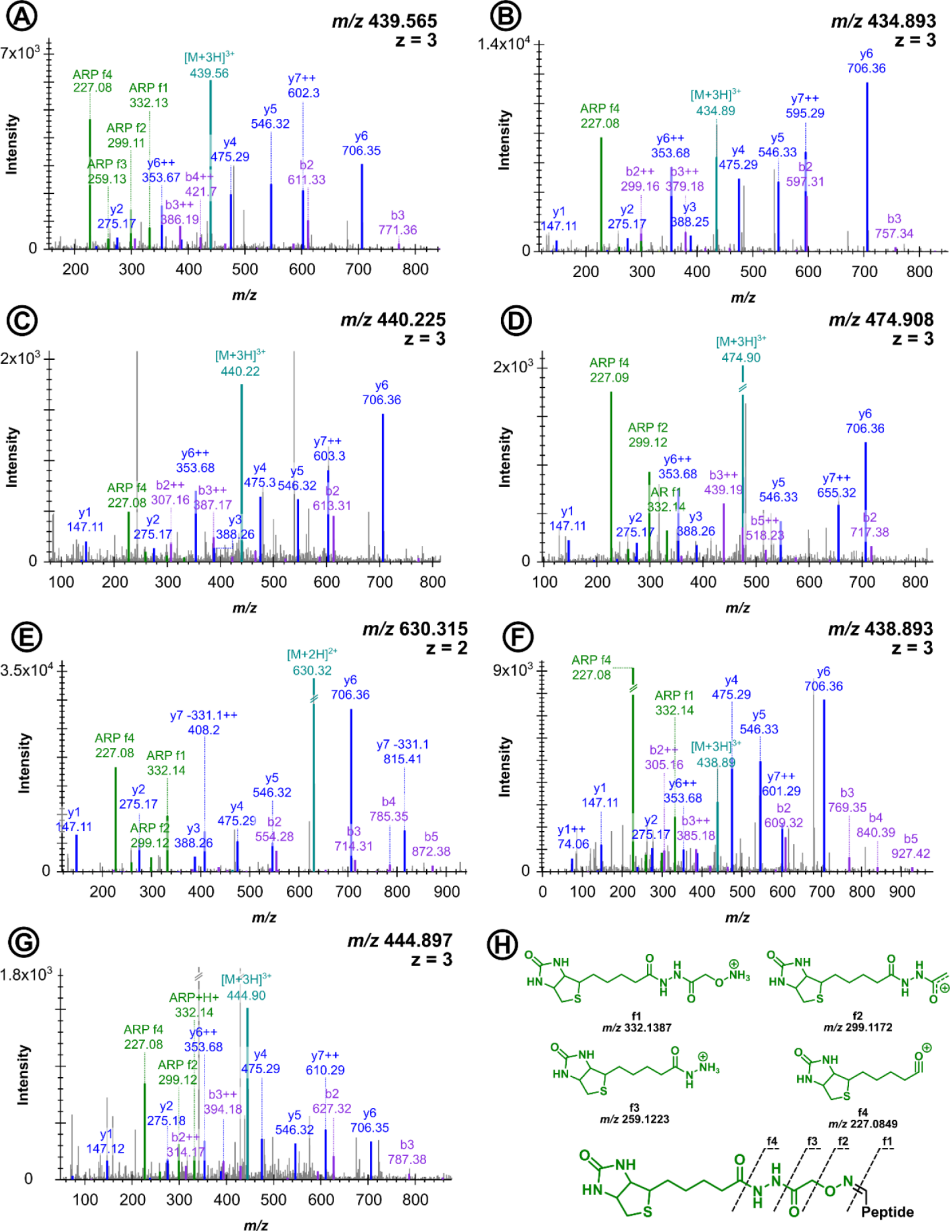
Tandem mass spectra of LKCASLQK (residues 222–229 in HSA) containing different ARP-derivatized carbonyl groups at Lys2. Mass shifts suggested acrolein Michael adduct (+369.147 Da, A), aldomine (+355.131 Da, B), glyoxal adduct (+371.126 Da, C), deoxyglucosone Michael adduct or Amadori rearrangement of hexose glycation (+475.174 Da, D), aminoadipic semialdehyde (+312.089 Da, E), malondialdehyde or methylglyoxal Schiff base (+367.131 Da, F), and malondialdehyde or methylglyoxal (enol form) Michael adduct (+385.142 Da, G). Cys3 was carbamidomethylated (+57.021 Da). ARP reporter ions are labeled in green (proposed structures in H), b-signals in purple, and y-signals in blue.

**Table 2:**
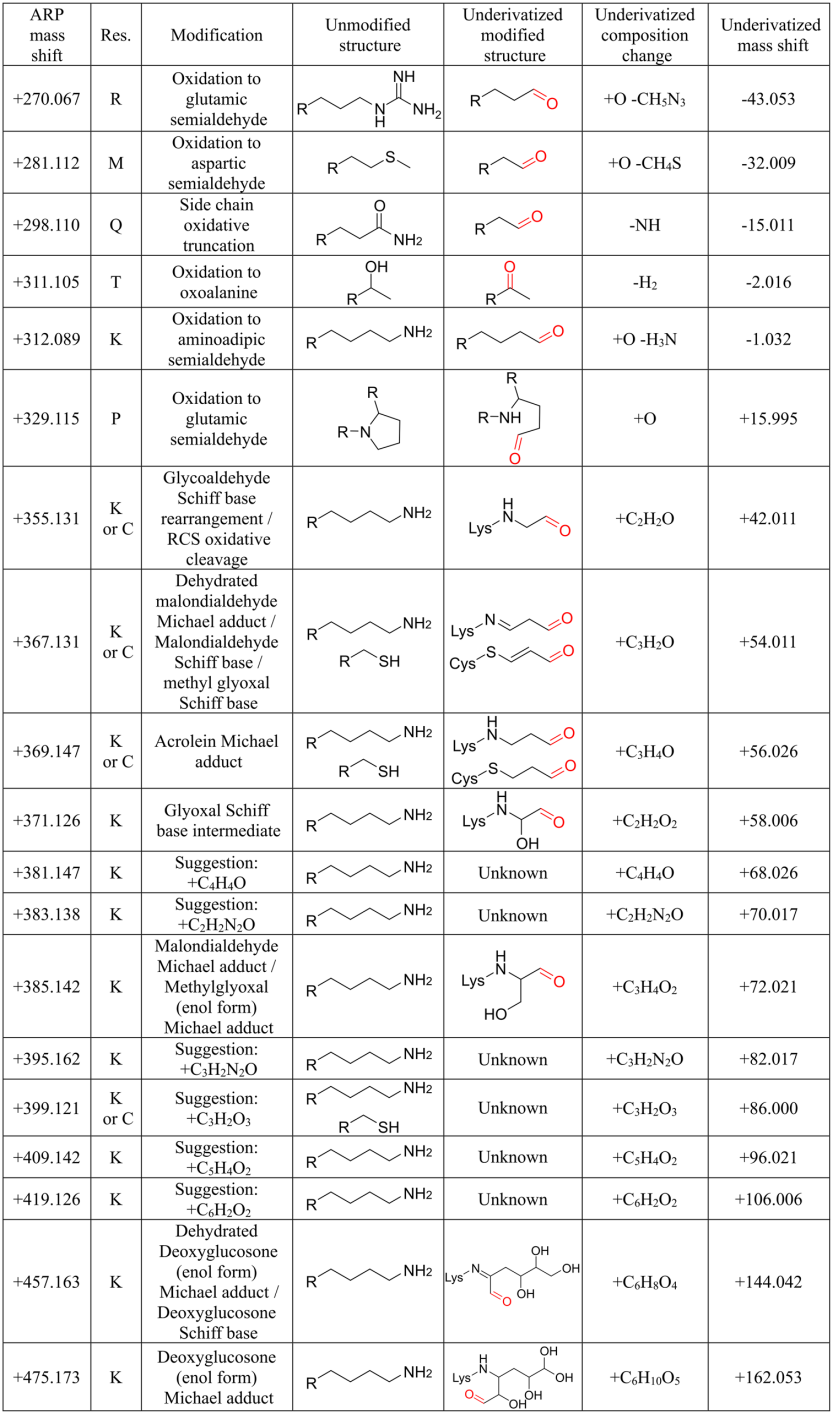
Mass Shifts and Proposed Structures of the ARP-Derivatized Reactive Carbonyl Modifications

The final set of confidently annotated ARP-derivatized HSA peptides contained 28 carbonylation sites that can be linked to metal-catalyzed side chain oxidation, including several residues proximal to known metal-binding sites.^33^ For example, Lys36 is proximal to the first metal-binding site comprising residues 25 to 27 of HSA (Asp-Ala-His), whereas Pro59, Thr103, and Thr107 are spatially close to the Cys58 metal-binding site (Figure S5). Furthermore, adducts with reactive carbonyl species generated by glycoxidation or lipid peroxidation were observed at different amino acids, with lysine being the most common (Figure 2 and Table 2). However, there were additional mass shifts that did not match the reported carbonyl PTMs considered in this study (Figure 2 and Table S6), although the sequence was confirmed by *de novo* sequencing, and the mass shift of the modification could be assigned to a specific residue. As we could not find modifications corresponding to these mass shifts in the literature, we proposed an elemental composition for these unknown modifications.

**Figure 2:**
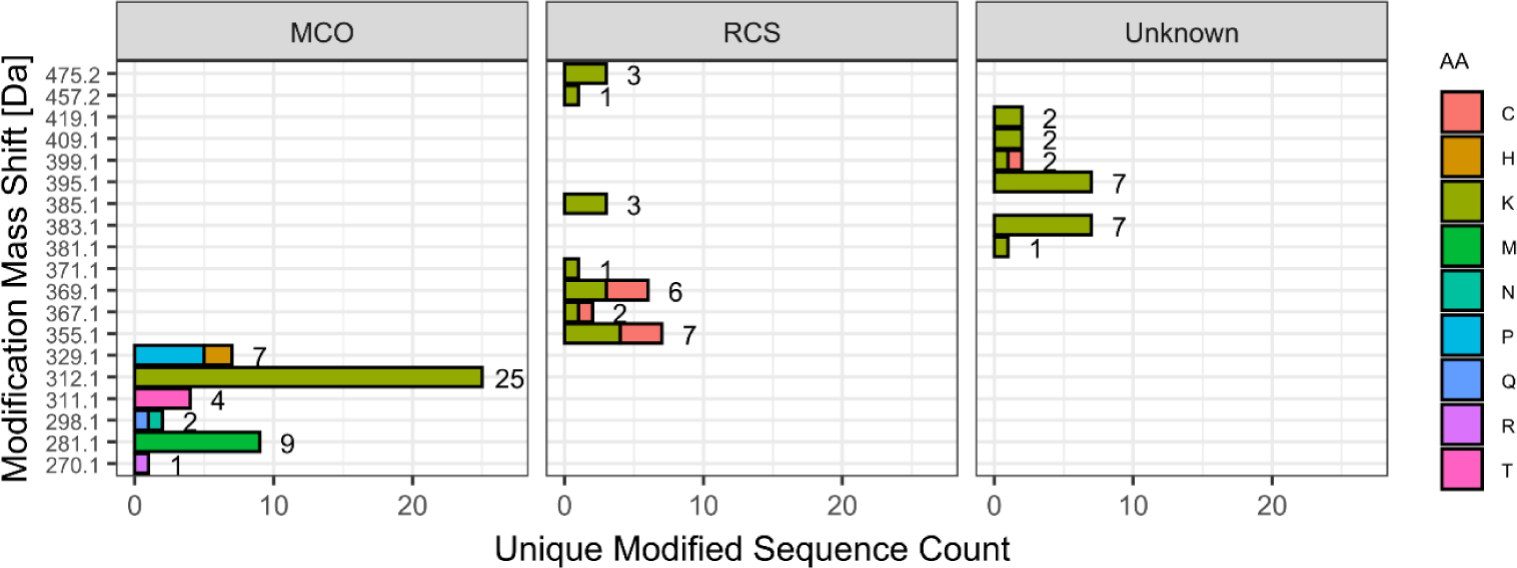
Classification of identified reactive carbonyl modifications. Mass shifts corresponding to ARP-labeled carbonyl groups are grouped based on inferred pathways: metal-catalyzed oxidation (MCO), adducts with reactive carbonyl species (RCS), or unidentified PTMs assigned by *de novo* sequencing (unknown).

ARP derivatization partially enabled the differentiation of modified isomeric peptides that are often separated by RP-HPLC but not distinguished by tandem mass spectrometry. For example, the glycoaldehyde-derived aldimine (−NH–CH_2_–CHO) of lysine residues is labeled with ARP, generating a mass shift of +355.1314 Da, which allows it to be distinguished from the nonreactive isomeric acetylation (−NH–CO–CH_3_).^34^ This mass shift was also observed exclusively on HSA peptides containing nucleophilic Cys58. Free cysteine residues are known to react with reactive carbonyl species (RCS), such as glycoaldehyde, glyoxal, and methylglyoxal, which can lead to protein inactivation.^35^ The observed mass shift might correspond to an analogous cysteine glycoaldehyde intermediate or a reactive carbonyl left after oxidative cleavage of adducts with larger free RCS. Other free reduced cysteines or those participating in cysteine bridges that had undergone *in vivo* reduction could generate these adducts, but no others were detected in this study. The aldimine (−NH–CHOH–CHO) generated by Schiff base formation with glyoxal^6^ can be distinguished from the isomeric carboxymethylated lysine (−NH–CH_2_–COOH). However, this was not always possible, as some isomeric modifications remained isomeric after derivatization. A mass shift of +475.174 Da specific to ARP-labeled lysine residues, indicating a mass shift of +162.053 Da for the underivatized modification, which suggests hexose glycation,^36^ but it remains unclear whether this adduct is generated by Schiff base formation of monosaccharides or by Michael addition of 1- or 3-deoxyglucosone (1 or 3DG). However, synthetic glucose-derived peptides are not derivatized by ARP (unpublished data) andthe ARP labeling of 1DG and 3DG appears to be more consistent with the observation of two closely eluting isomers of peptide K­[+475.174]­QTALVELVK (Figure S4). In some cases, derivatization made identification difficult. For example, two modifications, +385.142 Da and +367.131 Da, differed in their elemental composition by H_2_O, where the heavier modification predominantly lost water through in-source fragmentation. This was observed for Lys1 of the peptide K­[+367.131]­QTALVELVK (^549–558^HSA), which contained the isomeric Michael adducts with malondialdehyde^37^ or a methylglyoxal enol tautomer (both +72.021 Da), and K­[+385.142]­QTALVELVK, which contained Schiff base adducts with malondialdehyde^38^ or methylglyoxal^39^ (both +54.011 Da) (Figure S3). Most likely, Schiff base adducts were missed by the applied protocol because they were not stabilized by reduction. However, mass shifts due to in-source water losses may still occur, highlighting the need to consider modifications such as water or ammonia losses when annotating carbonyl modifications.

Most of the carbonylation sites were identified in HSA, mainly due to its high abundance in serum and its relatively long half-life of ∼19 days.^40^ Interestingly, only nine of the 59 Lys residues and the single reduced Cys residue present in mature HSA were carbonylated by reactive electrophiles (Table 3), whereas ten Lys residues were observed as aminoadipic semialdehydes, suggesting a high site specificity for carbonyl formation around a few nucleophilic amino acids. In particular, Lys223, located in subdomain IIA (or Sudlow site I) with high affinity for short-chain lipids and some drugs,^41–43^ was identified with 11 different reactive carbonyls. The most common carbonyl modification types on proteins other than HSA were MCO-induced side chain oxidation products at Lys, His, Met, and Thr, with the exception of the serotransferrin peptide C­[+367.131]­HLAQVPSHTVVAR (^260–273^serotransferin) with a modified Cys260, which was interpreted as a dehydrated malondialdehyde Michael adduct.

**Table 3:** Reactive Carbonyl Species and Modification Sites Identified in Human Serum Albumin as a Result of Reactive Electrophiles

Modification Site^a^	Distinct Modification Count	Modification Mass Shifts	Subdomain	Lipid-Binding Site
C58	3	+355.131, +369.147, +399.121^b^	IA	No
K214	3	+312.089, +369.147, +383.138^b^, +381.147^b^	IIA	Site 9
K219	4	+312.089, +383.138^b^, +395.138^b^, +409.142^b^, +457.163,	IIA	Site 8
K223	11	+312.089, +355.131, +367.131, +369.147, +371.126, +383.138^b^, +385.142, +395.138^b^, +399.121^b^, +409.142^b^, +419.126^b^, +475.174	IIA	Site 7^c^/Site 8^c^
K456	2	+312.089, +395.138^b^	IIIA	Site 9
K460	1	+395.138^b^	IIIA	Site 9
K543	3	+312.089, +383.138^b^, +395.138^b^	IIIB	No
K549	6	+355.131, +383.138^b^, +385.142, +395.138^b^, +419.126^b^, +475.174	IIIB	Site 5
K565	4	+312.089, + 369.147, + 383.138^b^, + 395.139^b^	IIIB	No
K588	1	+355.131	IIIB	No

a Modification site relative to the canonical sequence of HSA (Uniprot ID: P02768).

b Mass shifts corresponding to unknown ARP-reactive carbonyl species.

c Based on proximity, no defined electron density to map amino acid orientation.

### *De Novo* Sequencing Guided by Spectral Similarity and Missing Identifications

During validation of the DDA data and alignment of the chromatographic features of the DIA data sets, it was found that several unidentified peptides had the same fragment ions covering the unmodified part of the identified peptide sequences based on ion mobility and accurate mass. Assuming that the sequences of these peptides were identical and differed only in the PTM, it was possible to determine the mass shift of the unidentified PTM and predict its elemental composition, assuming that it could contain C, H, O, N, or S. This strategy is discussed for the peptide LK­[+312.089]­C­[+57.021]­ASLQK (*t*_r_ = 31.8 min) with Lys2 oxidized to aminoadipic semialdehyde (Table 2). Based on the mass of the y_6_-ion at *m*/*z* 706.355, representing the C-terminal sequence C­[+57.021]­ASLQK, and the related drift times of the precursor (3.32 ms) and the y_6_-ion (3.12 ms) within the known IMS offset range of UDMS^E^, an unidentified peptide with presumably the same C-terminal sequence eluted 1.6 min later (Figure 3A,B). In fact, *de novo* sequencing with PEAKS suggested the sequence LR­[+367.131]­C­[+57.021]­ASLQK with Arg modified as methyl imidazole formed with methyl glyoxal (MG-H). This sequence was not in the UniProt database but could be explained by a Lys223Arg mutation in HSA. However, this appears unlikely because the signal was detected in all 68 serum samples, while the corresponding unmodified sequence was not found. Therefore, another unaccounted modification at Lys2 seems more likely with a total mass increase of 395.089 Da after ARP derivatization, i.e., LK­[+395.137]­C­[+57.021]­ASLQK (Figure 3C), suggests a Lys modification with a mass increase of 82.02 ± 0.01 Da (Figure S6) corresponding to an elemental composition of C_3_H_2_N_2_O using ChemCalc.^44^ When this proposed modification was considered in PEAKS, an additional seven modification sites with a mass shift of +395.089 were identified in HSA but not in other proteins (Table 3).^42^

**Figure 3:**
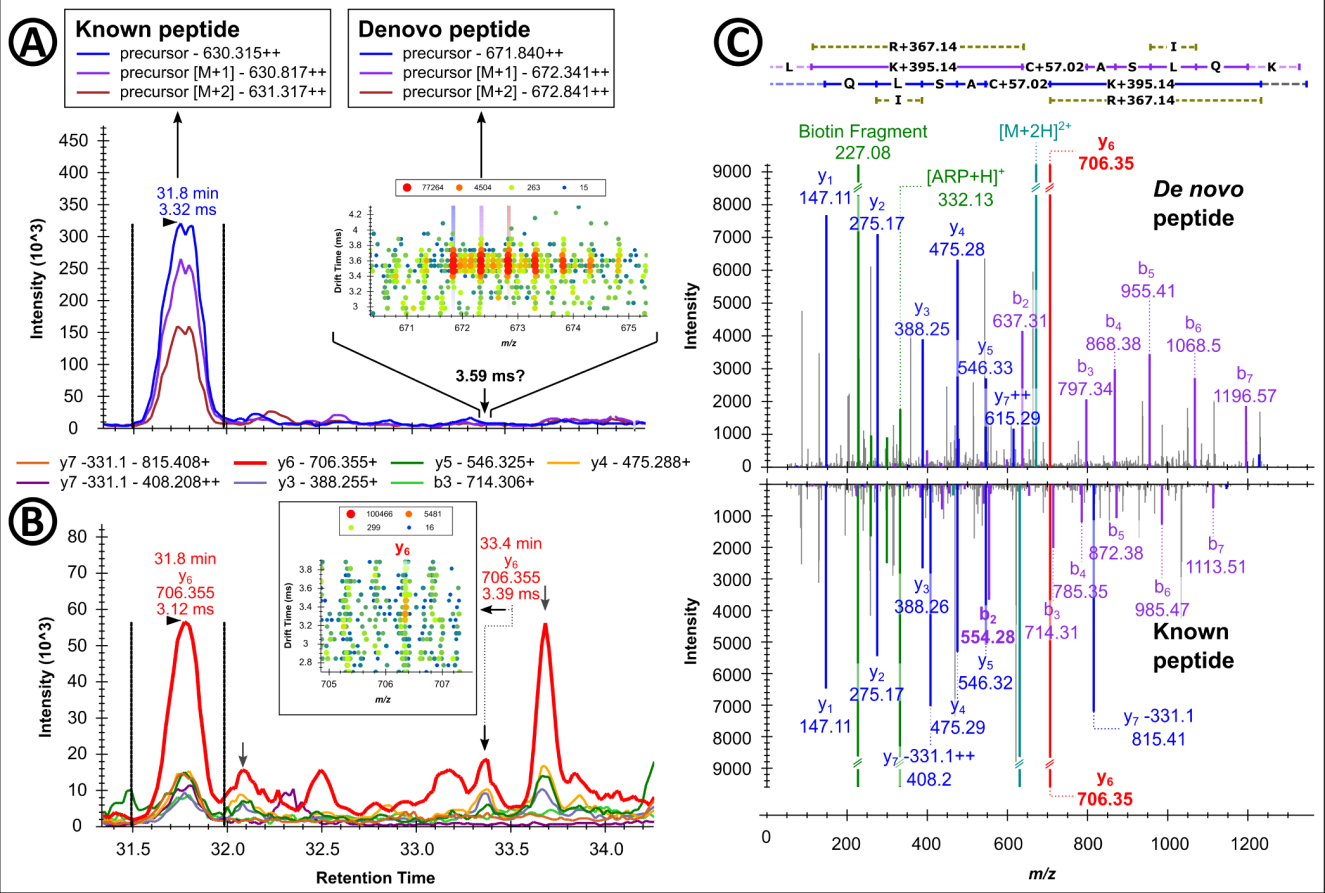
Identification of the HSA peptide LK­[+395.138]­C­[+57.021]­ASLQK based on the tandem mass spectra acquired for peptide LK­[+312.089]­C­[+57.021]­ASLQK. Extracted ion chromatograms (XICs) of the doubly protonated precursor ion (A) and selected b- and y-ion signals (B, y_6_ highlighted in red). Arrows indicate fragment ions shared by both peptides. The drift times of the precursor and y_6_ ions in IMS are given below the retention times. Mirror plots of the fragment ion spectra of the doubly protonated modified peptides (C). High-intensity signals are clipped and marked with double dashed lines.

By applying this strategy to other carbonylated peptides, further lysine residues with unknown mass shifts of +381.147 Da (1 residue), +383.138 Da (7 residues), +395.138 Da (7 residues), +399.132 Da (1 residue), +409.142 Da (2 residues), and +419.126 Da (2 residues), as well as one cysteine residue with a mass increase of +399.133 Da after ARP derivatization, were identified. These correspond to mass increases compared to the underivatized lysine residues of +68.026, +70.017, +82.017, +86.012, +96.021, and +106.006 Da, respectively, and +96.021 Da for cysteine (Table S6).

Considering these six unidentified modifications (Table S3), which most likely contain reactive carbonyls, 21 additional ARP-peptides could be added, resulting in a total of 86 validated carbonylated peptides. However, there were still fragment ion spectra displaying signals that may represent ARP-reporter ions, although the sequence could not be retrieved from the spectra, suggesting additional modifications with unaccounted mass shifts. When the data were processed with mgfHunter (https://github.com/ZhixuNi/mgfhunter), a tool designed to search fragment ion spectra for inspection of reporter ion signal patterns (see Supporting Information for details), 62,575 spectra were retrieved, of which 48.1% had no assigned PSM. This large number of unidentified spectra highlights the need to expand data interpretation strategies to discover physiologically relevant carbonylation PTMs. For example, by using DIA and IMS.

### Peptide-Centric Analysis of DIA Data Allows Detection of Carbonylated Proteins in RA and Control Serum Samples

Peptides were identified in the acquired DIA data using several peptide features, such as a constant ratio of precursor and fragment ion intensities along the chromatographic peak and drift time, indexed retention times (iRT), isotopic patterns based on elemental composition, and a similar intensity ratio of fragment ion signals in XICs and DDA PSMs. This allowed the detection of 86 ARP-peptides confidently identified in DDA mode in the UDMS^E^ data set collected for all 68 serum samples by retention time-aligned precursor ion signals. DDA results were aligned with DIA data using 10 ARP-peptides as endogenous iRT standards to correct for retention time drifts. The chosen ARP-derivatized peptides were identified confidently and detected in all of the EF-QC samples with high signal intensities. For additional confirmation of the aligned peptides, fragment ions were used when detected above the background, but in many cases, these signals were too weak. Furthermore, the integrated peak areas of the ARP-peptides had to be larger than the retention time-matched integration windows in the underivatized negative control samples (Figure S10). The ratio of the integrated area of an ARP-peptide to the corresponding area of the negative control (matrix background) was used to evaluate peptide detection (Figure 4). Considering all 6,732 precursor ion signals of the 86 confirmed ARP-peptides detected in all donor samples, 94.1% had a signal-to-background area ratio greater than 3, and 76.7% had a ratio greater than 10. Only the peptides ASS[−18.011]­AK­[+457.163]­QR (HSA), DTLM­[+281.112]­ISR (nonunique, IGHG1, IGHG2, IGHG3, or IGHG2), and SK­[+312.089]­EQLTPLIK (apolipoprotein A-II) could not be quantified at the precursor level (Table S7) due to strong integration interference from matrix background. Methods using the IMS of Synapt G2-S*i* have a reduced dynamic range due to detector saturation by abundant analytes.^45^ However, such limitations were not observed for ARP-peptides in this study, and IMS allowed lowering the detection limit of ARP-peptides by removing the integration interference of isobaric precursor ion signals, similar to a previous report.^19^

**Figure 4:**
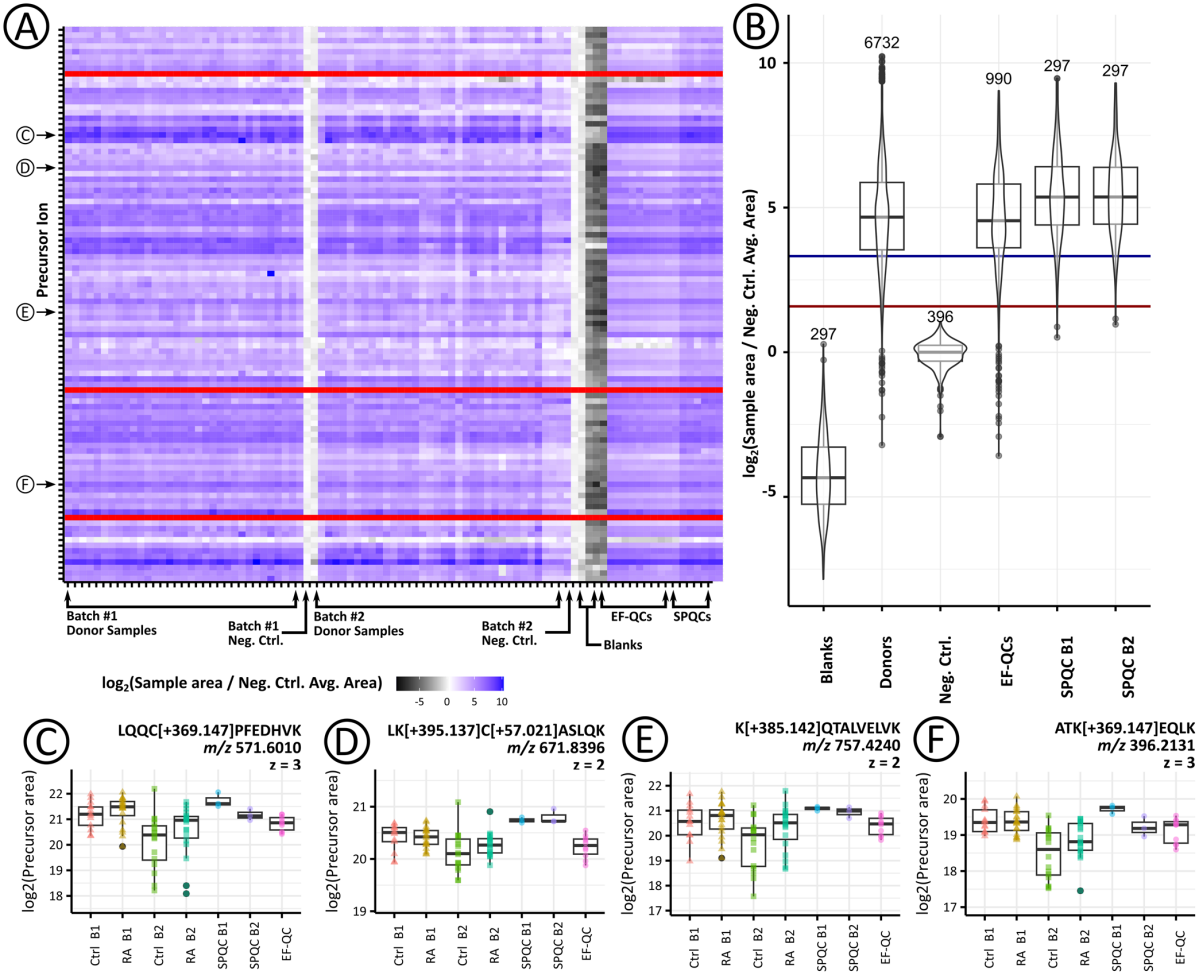
Peak areas obtained from the XICs of different ARP-derivatized peptides normalized to the corresponding region in the underivatized negative control (Neg. Ctrl.). Heatmap of the normalized peak areas of all 86 carbonylated peptides in all 68 donor and quality control serum samples (A) and box plots grouped by sample type (B). Peak area distributions are shown for selected precursor ion signals corresponding to the ARP peptides LQQC­[+369.147]­PFEDHVK (C), LK­[+395.137]­C­[+57.021]­ASLQK (D), K­[+385.142]­QTALVELVK (E), and ATK­[+369.147]­EQLK (F). Red horizontal lines in the heatmap correspond to precursor ions with integration interference that prevented detection; sample and precursor ion IDs are provided in Table S7. Log_2_-transformed ratios are used as a color fill gradient. The inflection point of the gradient corresponds to log_2_(3). In the box plots, dark blue and dark red lines represent the y-intercepts at log_2_(10) and log_2_(3), respectively.

As mentioned above, fragment ion signals of several carbonylated peptides represented by weak precursor ion signals were not detected in all serum samples, limiting their use for correct chromatographic peak selection. For example, the peptide SHC­[+57.021]­IAEVENDEM­[+281.112]­PADLPSLAADFVESK was detected only as a triply protonated ion based on the iRTs, while the fragment ion signals were not visible (Figure 5A; right panels). Only 30.3% of the integrated fragment ion signals had an XIC area that was at least 10-fold larger than the corresponding XIC area of the negative control (Figure S10). Although coaligned precursor and fragment ion signals were observed for other peptides with intense precursor ion signals, such as the triply protonated peptides ATK­[+369.147]­EQLK and K­[+355.131]­QTALVELVK (Figure 5B,C), there were significant challenges with automated chromatographic peak picking when multiple peptide variants were present in a sample. The Skyline base peak picking algorithm was biased by shared, intense fragment ions of other modified versions of the same peptide sequence that were detected at higher intensities, such as for peptide K­[+355.131]­QTALVELVK (Figure 5C). The triply protonated precursor ion signal of this peptide was misaligned with the identical fragment ion signals at *m*/*z* 872.545, *m*/*z* 771.498, *m*/*z* 700.460, *m*/*z* 587.376, and *m*/*z* 488.308 at ∼40 min, which also correspond to the y_4_ to y_8_ signals of the coeluting peptide QTALVELVK. The nonrandom nature of these common fragment ion signals also presented a challenge in the training of a useful mProphet peak picking model for automated peak selection. However, note that careful inspection of the peptide iRT and aligned precursor ion signals allowed for their manual correction, highlighting the importance of collecting precursor-level information for modified peptides and the need for software that allows for curation by experienced analysts. The short cycle time of 1.2 s in the UDMS^E^ method applied here allowed reasonable quantitation of 99.8% of all integrated precursor ion signals with at least nine data points along the peak, ensuring more reliable LC peak profiling, which has been shown to keep artifact integration deviations below 2%.^46^ In contrast, only 57.3% of the signals obtained in DDA mode were covered by at least nine data points along the peak (Figure S8). Thus, HD-DDA Trap mode was best for the identification of diverse ARP-labeled peptides, especially for weak signal intensities; HD-DDA Transfer with preference lists supported IDs of peptides producing intense multiply charged ions; and UDMS^E^ was best for peak integration.

**Figure 5:**
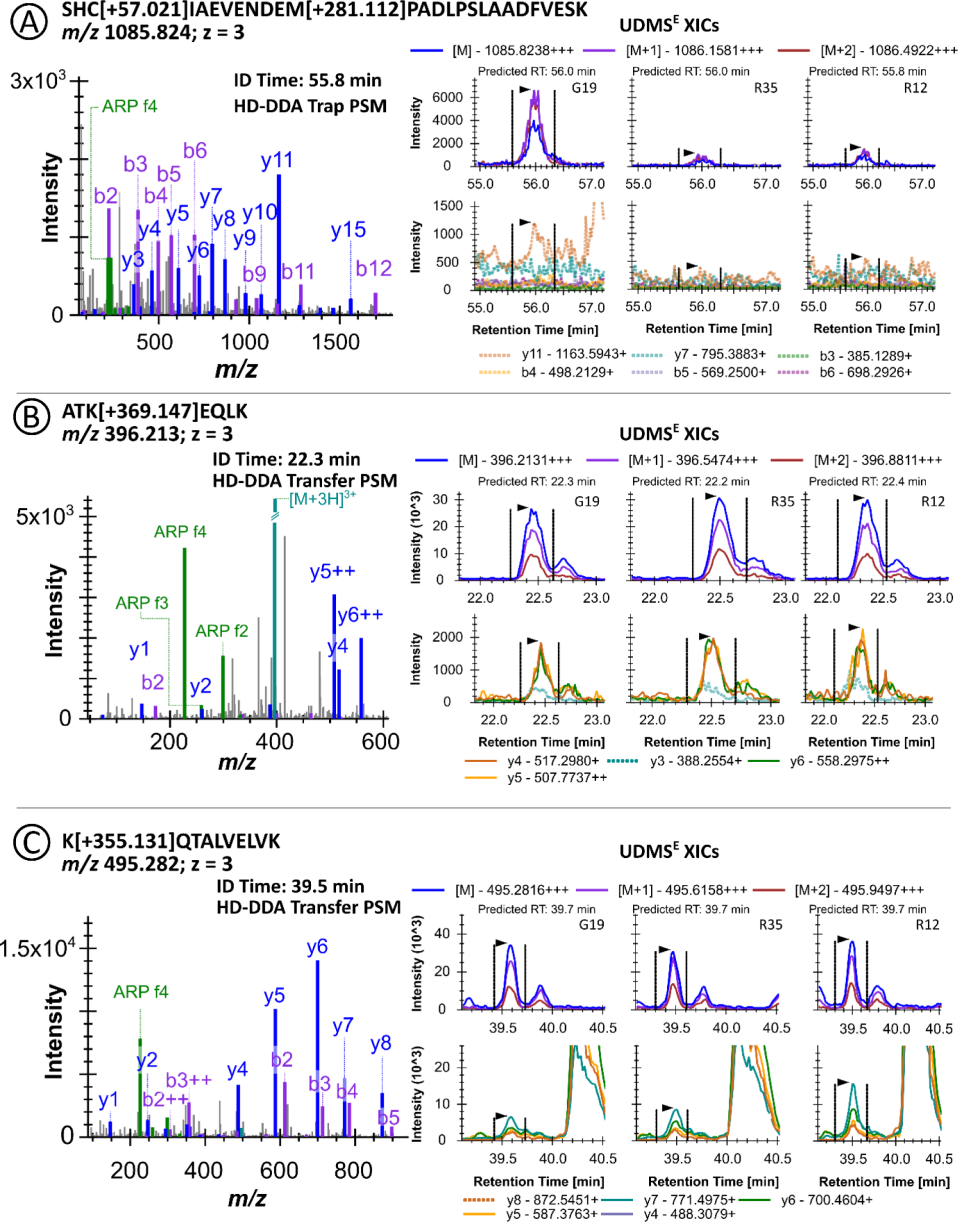
Best PSMs and XICs obtained by DDA and UDMS^E^, respectively, for the triply protonated ions of peptides SHC­[+57.021]­IAEVENDEM­[+281.112]­PADLPSLAADFVESK (^311–337^HSA) (A), ATK­[+369.213]­EQLK (^563–569^HSA) (B), and ATK­[+369.213]­EQLK (^563–569^HSA) (C). Representative XICs are shown for RA donor samples R12 and R35 and the control donor sample G19. For each peptide, XICs are shown for the first three isotopes of the precursor ion signals (top panels) and the five most intense fragment ion signals (bottom panels) selected from the DDA spectral libraries. Traces that could not be quantified due to integration interferences are shown as dashed lines.

With the identification and quantitation strategies established, the reproducibility of sample preparation for ARP-derivatized peptides in affinity-enriched samples was evaluated using the SPQCs. Considering the integrated precursor ion signals of all 86 ARP-peptides with area coefficients of variation (CVs) less than 20%, the intrabatch reproducibility was 79.8% for Batch #1 and 75.3% for Batch #2 (Figure S11), whereas the interbatch reproducibility was 65.9%. The EF-QCs measured were used to evaluate signal stability across the acquisition queue, which revealed that the peak areas significantly decreased in the second batch, typically by 21.8% (Figure S12). Considering both batches, only 42.7% of the precursor peak areas of the EF-QC samples had CVs below 20%, whereas 76.1% of the precursor areas of the EF-QCs in Batch #1 were below the 20% threshold, suggesting significant signal decay in the Batch #2 samples measured immediately after Batch #1 samples, i.e., 76 h after starting Batch #1. Statistical analysis using MSstatsPTM^30^ was done considering batch effects in the combined batches and in Batch #1 only. To compare the average of the adjusted modified peptide areas between RA and control donors, the null hypothesis was not rejected. The adjusted intensities of carbonylated peptides were similar between the RA and control cohorts, with no average means differing by more than 2-fold that could be separated with a 0.05% confidence of false rejection (Figure S13 and Table S8). Peak area box plots of the precursor areas showed a large variation between cohorts (Figures 4C–F). However, the signal scatter is unlikely to be due to sample preparation, as the SPQCs prepared in parallel contained only a small distribution with high reproducibility between both batches. It is more likely that these differences reflect individual distributions among the serum samples. Nevertheless, the observation of comparable adjusted intensities for carbonylation sites in both RA and control donors suggests that the modifications studied, such as MCO products and adducts with acrolein, malondialdehyde, glyoxal, methylglyoxal, and the proposed unknown RCS adducts, are ubiquitous in individuals with similar baseline levels unaffected by RA, at least within the precision of the current study based on a limited number of samples.

## Discussion

### Detection of Carbonylation Sites in Human Serum

The presence of multiple HSA carbonylation sites at similar levels in both donor and control samples suggests a basal level of modification that might reflect oxidative stress defense mechanisms. HSA has an antioxidant role^47^ through multiple mechanisms: 1) the predominant abundance in plasma with the free thiol group at Cys58 makes it the major protein scavenger for ROS in plasma;^3,48^ 2) the binding of transition metals^33^ to Cys58 minimizes MCO oxidation on other proteins but sequesters oxidation around the metal-binding sites, as observed here; and 3) the presence of several highly nucleophilic sites allows it to scavenge RCS.^49,50^

The modification profile of HSA observed here is in good agreement with seminal *in vitro* experiments, in which HSA was incubated with a variety of RCS, including methods targeting adducts at Cys58. For example, when a plasma sample was incubated with 4-hydroxy-2-nonenal (HNE), the most susceptible residues included Cys58, Lys219, Lys223, and Lys 549.^50^ Although no HNE adducts were observed here at these residues, they were carbonylated by multiple MCO and RCS, especially Lys223, with 11 different modifications detected. In contrast, incubation of HSA with MDA *in vitro* generated 6 modification sites, with Lys549 as the preferential site.^51^ In another *in vitro* study, 44 residues were modified by MDA, including Lys549.^37^ Interestingly, neither of these studies reported Lys223 as modified, although we detected it here in human plasma. HSA incubated with acrolein *in vitro* induces multiple modification sites, including Cys58.^52^ Similarly, acrolein adducts at Cys58 were also identified in an *in vitro* study using water-soluble cigarette smoke extracts.^53^ Both studies also reported modifications at Lys549, a hotspot for reactive electrophiles reported here, but missed the acrolein Michael adduct at Lys565 observed in the present study. Acrolein Michael adducts at Cys58 were detected *in vivo* in blood samples collected during the reperfusion stage of liver hepatectomy by an adductomics workflow.^54^ Finally, numerous *in vitro* and *in vivo* studies characterizing glycation sites in HSA have been compiled,^55^ highlighting Lys223 and Lys549 among the most frequently reported sites—both of which were identified in this study as advanced glycation end-products (AGEs) of glycoaldehyde and deoxyglucosone.

The low levels of protein carbonylation *in vivo* greatly complicate their detection. There are only a few reports on human plasma using different carbonyl-specific derivatization reagents that allow their affinity enrichment but at the same time complicate the comparison of reported data sets (see Supporting Information of Havelund et al.).^16^ Therefore, the following discussion focuses on recent reports using biotinylated probes, peptide-level enrichment, and LC-MS/MS studies deposited in public repositories such as PRIDE or Panorama. All carbonylation hotspots identified here (Table 3) were observed as at least one ARP-derivatized carbonyl PTM in human plasma from a healthy donor in a previous study from our laboratory.^20^ The detection of these modification sites in all serum samples of a larger cohort (*n* = 68) further supports the endogenous prevalence of these modification types in human plasma. In a similar study, Havelund et al. identified multiple MCO-generated carbonylation sites in human plasma proteins derivatized with long-chain biotin hydrazide (lc-BHZ) after tryptic digestion and avidin-based enrichment of the derivatized peptides by LC-MS.^16^ They identified carbonylation sites Lys28, Lys36, Pro137, Arg210, Lys214, Lys375, Lys456, Thr520, Lys565, and Met572 in HSA, which were confirmed here (Table 1). It should be noted that a larger set of MCO carbonyl products has been observed for HSA and BSA oxidized *in vitro*.^16,20^ This suggests that the MCO sites observed by Havelund et al. and here may represent oxidation-prone sites of serum albumin under physiological conditions. It is likely that additional modification sites will be identified *in vivo* by more sensitive methods. Overall, considerable discrepancies remain between the specific modification sites generated *in vitro* and those detectable *in vivo* by a diverse set of reactive electrophiles and MCO. Nonetheless, the consistent identification of the most reactive residues toward reactive carbonyl species (RCS) across studies is encouraging, and several of these sites are corroborated by the *in vivo* findings of this study in a moderately sized cohort of 68 individuals. Moreover, the methodology presented here is capable of capturing signals of carbonyl modifications generated by multiple pathways enhanced through excessive oxidative stress, allowing a more comprehensive analysis of proteome-wide changes instead of focusing on single modification types.

Havelund et al. reported 40 carbonylated proteins, 25 more than in the current study, partly due to higher sample amounts (3.5 mg protein versus 2.0 mg protein per sample)^16^ and higher loadings on LC-MS, i.e., 100% of the eluate of the affinity enrichment compared to 5% in the current study. However, at these 35-fold higher sample loads, we observed sample carryover and a steady increase in column backpressure, resulting in unstable chromatographic conditions and poor reproducibility of precursor areas, which was also observed by Havelund et al. (see Supporting Information for reanalysis details). Only 1.4% of the lc-BHZ-derivatized carbonylated peptides had XIC area CVs below 20%, and the median CV was 57.9% (Figure S14; Skyline document with reanalysis of PXD002966 available at https://panoramaweb.org/HumanSerumCarbonylationRA.url). In the current study, the integrated areas of the SPQC samples suggested good reproducibility of sample preparation and analysis for up to 45 samples. However, the fraction-specific quality control samples revealed methodological challenges for larger acquisition queues that need to be addressed for clinical studies. In this regard, a standard of an oxidized and derivatized protein that is not homologous to human serum proteins may allow the normalization of the analysis of affinity-enriched samples in larger cohorts. We suspect that chromatographic instability may be caused by difficult-to-detect monomeric avidin impurities released during peptide elution and a high background of underivatized peptides present in the enriched fractions due to nonspecific interactions. In previous work, we have shown that ultrafiltration efficiently removes the avidin monomer,^20^ but it may still be present at low levels, contaminating RP-columns over time when many samples are analyzed. Avidin could also be replaced by streptavidin, which may reduce the nonspecific background and thus allow higher sample loads on the column, potentially improving sensitivity without disturbing chromatographic stability. The current workflow identified carbonylation sites mainly in abundant plasma proteins, while medium- and low-abundance proteins were mostly missed. As the search for RA-specific markers was limited to a few proteins, future studies should analyze samples depleted of abundant proteins to search for markers in proteins that may be more relevant to the pathology of RA or focus on subcomponents of plasma, such as extracellular vesicles, although this will significantly limit sample throughput.^56^ The increased complexity of sample processing will require critical evaluation of the recovery rates of carbonylated peptides and their valid quantitation. Despite its limitations, the established protocol demonstrated good reproducibility and provides a reliable benchmark for carbonylation site identification, with potential for even better performance in samples with narrower protein dynamic ranges, such as tissues or cell cultures.

### Expansion of Search Strategies for Carbonylation Site Identification

Most publications targeting carbonylation sites have relied on derivatization tags and closed search database matching software, such as SEQUEST^57^ and Mascot.^16,29,58,59^ Some researchers have noted that tag-related intense reporter ions penalize the scoring of PSMs with Mascot and have suggested removing these reporter ions from the fragment ion spectra.^16,29^ Although this approach improves scoring, we prefer not to remove relevant signals from mass spectra. Instead, we advocate tailoring tools to identify the reporter ions and use these signals and signal ratios to enhance the score obtained from the peptide backbone fragment ion signals, which should remain the major contributor to the final score. Therefore, we tested the PEAKS search engine, which is more tolerant of signals not generated by peptide backbone fragmentation, including ARP-reporter ions, because it relies on *de novo* sequencing prior to database matching. Recently released versions of PEAKS (v11 and later) allow the inclusion of modification-specific reporter ions for improved identification of modified and derivatized peptides. Nevertheless, we suspect that most search engines have not been validated to properly control FDR for rarely studied derivatized carbonylated peptides. This may also explain why only 86 out of the 575 ARP-derivatized carbonylated peptides proposed by PEAKS at a 1% FDR level could be confirmed after excluding 324 peptides likely arising from Asn/Gln and Asp/Glu side-product artifacts from the original total of 899. Although automated peptide identification is an essential step in the processing of large LC-IMS-MS/MS data sets, we recommend that carbonyl-derivatized peptides identified by database search engines should be considered only as a preliminary indication, requiring manual validation. In general, new identification strategies for physiologically relevant carbonyl PTMs should be explored. Tools capable of open mass searching (OMS) could be investigated, especially MSFragger,^60^ as newer iterations allow the use of reporter ions to adjust scoring. OMS strategies have already proven useful in characterizing a wide range of oxidative modifications with relatively small mass shifts (∼-40 to 80 Da).^61^ However, the much larger mass shifts of the ARP-derivatized carbonylation sites might complicate the accurate annotation of modification sites, which warrants further investigation.

The results presented here suggest that DIA may provide an advantage over DDA with spectrum-centric analysis, which has typically been used for the identification and detection of carbonylated peptides.^16,29,57^ Due to the diversity of reactive carbonylation sites and their low physiological levels, DDA methods are important in PTM profiling since they can generate minimally chimeric fragment ion spectra that can be searched within a large search space. However, we consider that the combination of DDA-based database searches and validation using additional peptide-specific features of the modified peptides is essential. Significant achievements in data compatibility through open-source tools such as Skyline^21,22^ allow for the evaluation of data sets with a variety of search engines, including SEQUEST, Mascot, and PEAKS PTM, which are often used in previous carbonylation studies. Moreover, this platform enables the integration of DDA and DIA data to create comprehensive spectral libraries and DIA data acquisition schemes to optimize LC-MS methods with shorter cycle times, improved quantitation of weak signals, and an unbiased collection of fragment ion data of query peptides in all samples. As can be expected for complex matrices with sample-to-sample variation, such as human plasma samples, reasonable fragment ion XICs were not obtained for all samples. Consequently, precursor ion XICs and iRTs of the corresponding peptides proved critical for evaluating peptide detection.

## Conclusion

The use of biotinylated, carbonyl-specific derivatization probes enabled the identification of a diverse set of modifications originating from multiple sources associated with excessive oxidative stress, MCO, AGEs, and ALEs. The combination of DDA and DIA methods facilitated both the identification and robust quantitation of 86 carbonylated peptides in serum samples from healthy individuals (*n* = 29) and patients diagnosed with rheumatoid arthritis (*n* = 39), of which 75 were located in HSA. HSA was the main nucleophile, with major modification sites at Cys58, Lys214, Lys219, Lys223, Lys456, Lys543, Lys549, Lys565, and Lys588, including five sites located in lipid-binding domains of HSA. Interestingly, 11 different reactive carbonyls were identified at Lys223, which is located in subdomain IIA, also known as Sudlow binding site I. Spectral similarity and DIA proved useful in identifying unannotated peptide features of interest that shared diagnostic fragment ions, while IMS enabled the identification of the precursor of origin by using precursor and fragment ion alignment. The set of *de novo* sequenced PTMs presented here underscores the need to incorporate open mass search strategies in the future to characterize PTMs generated by reactive carbonyl species that have not been considered or reported. The identified carbonylated peptides were ubiquitously detected in healthy individuals and patients diagnosed with rheumatoid arthritis. Although the area CVs were less than 20% for at least 75% of the carbonylation sites in each batch, there was no evidence of any disease-related up- or downregulation of the most abundant serum proteins. Most likely, the identified residues represent regular antioxidant mechanisms, with HSA scavenging many ROS, AGEs, and ALEs in blood. Further research is needed to improve the reported strategy in terms of sensitivity, which will enable the identification of more carbonylation sites in other proteins and enhance robustness to quantify carbonylation sites in large serum sample sets.

## Supplementary Material





## Data Availability

The mass spectrometry proteomics data have been deposited to the ProteomeXchange Consortium via the Panorama Public partner repository with the data set identifier PXD058666 (DOI: 10.6069/w1vq-ft51). Additionally, curated Skyline documents and R scripts used in this study can be found at https://panoramaweb.org/HumanSerumCarbonylationRA.url.

## Notes

Additional experimental details, summary of reanalysis of data submitted with proteomeXchange identifier PXD002966, experimental design of the present (Figure S1), overlap of identified precursor ions and peptides carrying ARP-derivatized modifications based on DDA results (Figure S2), precursor ion signal and fragment ion spectra of the triple protonated HSA peptide K­[+385.142]­QTALVELVK and ambiguous peptide K­[+385.142]­QT[−18.011]­ALVELVK or K­[+367.131]­QTALVELVK (Figure S3), precursor ion signal and fragment ion spectra of the triple-protonated HSA peptide K­[+475.174]­QTALVELVK (Figure S4), carbonylation sites identified at Gln57, Pro59, Thr103, and Thr107 near the metal ion binding site Cys58 are highlighted as a stick representation (Figure S5), likely fragmentation scheme of the double-protonated HSA peptide LK­[+395.137]­C­[+57.021]­ASLQK (residues 222–229) Figure S6), histograms showing the signal intensities of ARP-reporter ions (Figure S7), distribution of the number of data points over the precursor signal in XICs (Figure S8), examples of correctly and falsely identified ARP-labeled peptides (Figure S9), fragment peak areas calculated from the XICs of 86 ARP-derivatized peptides normalized to the corresponding retention time region in the underivatized negative control (Figure S10), histograms of the coefficient of variation (CV) of peak areas of ARP-labeled peptides in XICs (Figure S11), signal distribution of ARP-peptides along the analysis time line (Figure S12), volcano plots for differential analysis adjusted intensities of ARP-peptides using MStatsPTM (Figure S13), coefficient of variation (CV) of the precursor area of peptides derivatized with long-chain biotin hydrazide (lc-BHZ) (Figure S14) (PDF)
Bradford protein concentrations (Table S1), clinical sample batches (Table S2), list of modifications searched in the current study (Table S3), PEAKS identification summary: 1% FDR at the peptide level (Table S4), confirmed carbonylated proteins and peptides (Table S5), calculation of unknown PTM offsets (Table S6), precursor areas and signal-to-matrix background ratios (Table S7), MSstatsPTM adjusted model (Table S8), DDA universal spectrum identifiers (USI) (Table S9) (XLSX)

## #

Center for Proteomics and Metabolomics, Leiden University Medical Center, 2333 ZG Leiden, The Netherlands

## Abbreviations

ARP: Aldehyde reactive probe
CVs: coefficients of variation
DDA: data-dependent acquisition
DIA: data-independent acquisition
EF-QC: enriched fraction quality control
HAS: human serum albumin
IMS: ion mobility spectrometry
iRT: indexed retention time
LC-MS/MS: liquid chromatography coupled to tandem mass spectrometry
MCO: metal-catalyzed oxidation
NEF-QC: nonenriched fraction quality control
PSM: peptide spectrum match
RA: rheumatoid arthritis
RCS: reactive carbonyl species
SPQC: sample preparation quality control
XIC: extracted ion chromatogram
