# Supplementary material for: Screening of Protein Carbonylation Sites in Human Serum by Ion Mobility Mass Spectrometry
Source: J Proteome Res. 2025 Jun 14;24(7):3412–28. doi: 10.1021/acs.jproteome.5c00093 (PMC12235708; doi:10.1021/acs.jproteome.5c00093)
Supplement: Supplementary file 1 [file pr5c00093_si_001.pdf]

Supporting Information

Screening of protein carbonylation sites in human serum by ion mobility mass spectrometry

Juan Camilo Rojas Echeverri[1,2], Sanja Milkovska-Stamenova[1,2], Ulf Wagner[3], Ralf Hoffmann[1,2]

[1]Institute of Bioanalytical Chemistry, Faculty of Chemistry, Universität Leipzig, 04103 Leipzig, Germany; [2]Center for Biotechnology and Biomedicine, Universität Leipzig, 04103 Leipzig, Germany, [3] Division of Rheumatology, Department of Endocrinology, Nephrology, Rheumatology, Universität Leipzig, 04103 Leipzig Germany

Table of contents

Additional experimental details.......... S2

Summary of re-analysis of data submitted with proteomeXchange identifier PXD002966 .... S5

Figure S1: Experimental design of the present study.......... S6

Figure S2: Overlap of identified precursor ions and peptides carrying ARP-derivatized modifications based on DDA results.......... S7

Figure S3: Precursor ion signal and fragment ion spectra of the triply protonated HSA peptide K[+385.142]QTALVELVK and ambiguous peptide K[+385.142]QT[-18.011]ALVELVK or K[+367.131]QTALVELVK.......... S8

Figure S4: Precursor ion signal and fragment ion spectra of the triply protonated HSA peptide K[+475.174]QTALVELVK.......... S9

Figure S5: Carbonylation sites identified at Gln57, Pro59, Thr103, and Thr107 near the metal ion binding site Cys58 are highlighted as a stick representation.......... S10

Figure S6: Likely fragmentation scheme of the double-protonated HSA peptide LK[+395.137]C[+57.021]ASLQK (residues 222-229).......... S11

Figure S7: Histograms showing the signal intensities of ARP reporter ions.......... S12

Figure S8: Distribution of the number of data points over the precursor signal in XICs.......... S13

Figure S9: Examples of correctly and falsely identified ARP-labeled peptides.......... S14

Figure S10: Fragment peak areas calculated from the XICs of 86 ARP-derivatized peptides normalized to the corresponding retention time region in the underivatized negative control.......... S15

Figure S11: Histograms of the coefficient of variation (CV) of peak areas of ARP-labeled peptides in XICs...... S16

Figure S12: Signal distribution of ARP-peptides along the analysis time line.......... S17

Figure S13: Volcano plots for differential analysis adjusted intensities of ARP-peptides using MStatsPTM....... S18

Figure S14: Coefficient of variation (CV) of the precursor area of peptides derivatized with long-chain biotin hydrazide (lcBHZ).......... S19

References S20

Additional experimental details

Data dependent acquisition settings

For measurements performed in DDA mode, TWIMS was used to separate either precursor or fragment ions with the HD-DDA Transfer and HD-DDA Trap methods, respectively. Due to instrumental requirements, there are some differences between the HD-DDA methods used in terms of the *m/z* scan range used and the IMS settings required to obtain optimal ion mobility separation. In addition to the initial profiling for pooled samples, these were reanalyzed using an exclusion list of the top 100 precursor ions identified (based on intensity) and preference lists to obtain better spectra of ARP-derivatized precursor ions that did not have optimal peptide spectrum matches (PSMs), either due to under-fragmentation or overall precursor abundance, requiring longer scan times.

HD-DDA transfer was based on mass spectra acquired from *m/z* 300 to 1800 and tandem mass spectra acquired from *m/z* 50 to 2000 in resolution mode (R = 20,000 at *m/z* 400; FWHM) using an MS scan time of 0.2 s. Product ion scans were triggered at signal intensities above 1000 counts and were acquired once for up to 100,000 counts for a maximum time of 0.8 s. Fragmentation was induced in the post-TWIMS transfer cell using collision energy ramps from 12.3 to 17.8 V (start/end) at *m/z* 300 to 81.5 to 98.4 V (start/end) at *m/z* 1800. Tandem mass spectra were triggered for the three most intense signals using a dynamic exclusion window of ±250 mDa for 15 s, a full TWIMS cycle ramped wave velocity of 500 to 1200 m/s (start to end) and wave height of 40 V, pre-IMS trapping at 15 V for 500 µs, 0 V extraction, and an IMS delay of 1000 µs after trap release.

HD-DDA Trap was based on mass spectra acquired from *m/z* 300 to 1800 and tandem mass spectra acquired from *m/z* 50 to 5000 in resolution mode (R = 20,000 at *m/z* 400; FWHM) using an MS scan time of 0.2 s. Product ion scans were triggered at signal intensities above 1000 counts and were acquired once for up to 100,000 counts for a maximum time of 0.8 s. Fragmentation was induced in the pre-TWIMS trapping cell using collision energy ramps from 12.3 to 17.8 V (start/end) at *m/z* 300 to 81.5 to 98.4 V (start/end) at *m/z* 1800. Tandem mass spectra were triggered for the five most intense signals using a dynamic exclusion window of ±250 mDa for 15 s, a full TWIMS cycle ramped wave velocity of 2500 to 400 m/s (start/end) and wave height of 40 V, pre-IMS trapping at 15 V for 500 µs, 0 V extraction, and an IMS delay of 1000 µs after trap release[1,2]. The pusher interval for incoming fragment ions was synchronized for the HD DDA trap as previously reported[1] and according to Waters standard operating procedures to generate a "wideband enhancement" (WbE). Briefly, the ion detected at *m/z* 785.842 was fragmented at 35 V in the trap cell and the resulting fragment ions were separated by TWIMS. The recorded ion mobility spectrum was processed using Mass-Lynx V4.2 SCN983 and DriftScope v2.9 to generate an *m/z* drift time calibration file based on the distribution of singly

charged fragment ions. HD-DDA trap experiments can be performed without WbE, but here we always refer to HD-DDA trap with WbE.

Skyline data filtration steps

Data import and validation in Skyline relied heavily on a detailed protocol recently reported by our group (see Supporting Information).[3] Briefly, the Skyline data validation involved the following steps:

(1) Creation of a Skyline document containing only the results of the HD-DDA data collected from the RQCs, considering only the extracted ion chromatograms (XICs) of the first three isotopes of the precursor ions, using TOF as the mass analyzer and a resolution of 20,000. XICs were generated within 5 minutes of the PSM identification times.

(2) Proposed peptides were verified based on the peptide sequence coverage of their PSMs and the presence of ARP reporter ions. In addition, the reproducibility of the ARP-derivatized peptide signals in the XICs was evaluated. For the analysis of the enriched fractions, all non-derivatized peptides were removed except for peptides corresponding to spiked DnaK and previously determined native indexed retention time (iRT) peptides.

(3) Import the fraction specific system suitability quality control (SSQCs) samples (i.e. EF-QCs and NEF-QCs) measured with UDMS^E and negative control samples (i.e. same matrix without ARP reagent) using the retention times of the PSMs from the DDA files as a reference for chromatographic peak selection (± 5 min). The top 6 fragment ions (based on PSM intensity) were selected from the DDA spectral libraries, considering the y- and b-ion series up to y_3 and b_3. The best scoring peptide was automatically selected based on its PEAKS -10logP score for peptides represented by more than one PSM. When a PSM was present in both HD-DDA spectral libraries, the HD-DDA Transfer spectral libraries were preferred for fragment ion target selection because the tandem mass spectra of these PSMs were more similar to those collected in the UDMS^E data.

(4) Define the drift time of the precursor ion in IMS and include it in an ion mobility library to generate ion mobility filtered XICs.

(5) Re-import the results from the SSQCs and negative controls measured with UDMS^E data with the defined filters using an IMS resolution of 20 and include negative control samples (same matrix without added ARP reagent).

(6) Review the precursor and fragment ion targets and flag as non-quantitative those that suffered from integration interference even after IMS filtration and for fragment ions that had no discernible chromatographic peak shape in any replicate and therefore did not match the chromatographic profile

of the precursor ions. For the enriched fractions, peptides that had matching chromatographic peaks in the negative controls were identified as false positives and removed from further analysis.

(7) Generate indexed retention time (iRT) calculators using iRT standards consisting of DnaK (non-enriched fractions) or endogenous ARP-peptides (enriched fractions). For the enriched fractions, previously identified ARP peptides under the ProteomeXchange identifier PXD023738 (also available at https://panoramaweb.org/ULeipzig_ARP_Protein_Carbonylation_Method.url) were defined as native iRT standards. For the non-enriched fractions, peptides from the spiked DnaK were used as iRT standards.

(8) All remaining peptides were indexed in the Retention Time Calculator after careful curation of the chromatographic peaks in the SSQC samples.

(9) Once precursor ion mobility and peptide iRTs were curated, all control and RA samples measured with UDMS^E were imported using the iRTs as a guide to generate XICs with a window of ± 5 min from the predicted retention time and applying IMS filtering with the built IMS library.

(10) Chromatographic peaks were manually curated and refined in Skyline documents for the non-enriched and enriched fraction data sets that are provided in https://panoramaweb.org/HumanSerumCarbonylationRA.url. Manual LC peak curation is extremely time consuming. Pre-target filtration, ion mobility library curation, and iRT indexing of curated peptide elution times were essential to manage the data curation of over 200 LC-MS/MS data sets acquired in this study.

Reporter ion detection with mgfHunter

Detection of fragment ion spectra containing ARP reporter ion signals relied on mgfHunter, a tool developed by Dr. Zhixu Ni available at https://github.com/ZhixuNi/mgfhunter. Raw TWIMS-DDA files were analyzed with PEAKS Studio and .mzXML exports were generated. These exports were converted to peak lists in mascot generic format (MGF) files using MSConvert (v 3.0.21332-77ab1c7) and fragment ions with intensities below 100 absolute unit counts were deleted. This intensity threshold was defined by manual inspection of ARP-peptide PSMs, where signals below this level were considered unreliable because their signal-to-noise ratio was < 3. The converted .mgf files and a list of reporter ARP fragment ions were used as input to mgfHunter to search for them with an *m/z* error tolerance of ± 25 mDa in all fragment ion spectra. The detection score assigned to each reporter ion was as follows:

Name	Monoisotopic mass	Ion type	Charge state	Ion score
a	227.0854	fragment	1	60
b	332.1387	fragment	1	25
c	299.1178	fragment	1	25
d	259.1223	fragment	1	25
nl	331.1314	neutral loss	1	5

A cumulative score of 85 was required for detection of the ARP reporter ion. These scores were determined based on validation of ARP-derivatized peptides where the singly charged fragment ion of the biotin moiety of ARP with *m/z* 227.0849 was observed in all confirmed peptides, but the presence and relative intensity of the other fragments were found to be modification specific as reported in previous work.[2] The other known reporter ion corresponding to the singly protonated ARP-moiety at *m/z* 331.1314 was not intensely observed in peptides LC[+57]TVAT[+311.1]LR, YK[+312.1]AAFTEC[+57]C[+57]QAADK, LDELRDEGK[+369.1]ASSAK, DEGK[+381.1]ASSAK, DEGK[+383.1]ASSAK, ASSAK[+383.1]QR, EC[+57]C[+57]EK[+312.1]PLLEK, K[+395.1]QTALVELVK, and ATK[+369.1]EQLK. However, these peptide spectra showed intense reporter ions at *m/z* 259.1223 and *m/z* 299.1178. Since detection based on a single fragment ion was considered unreliable, the minimum score detection was increased to rely on at least two ARP reporter ions. These filtered MS/MS scans were then compared with the PSMs identified by PEAKS (results filtered at 1% FDR at the peptide level), and the final list of confidently annotated ARP-peptides. This was done by checking the scan number (e.g. scan 2845 of file S4_20_20_11.mzXML) annotated by each of these tools and evaluating the overlap in R (documented in the R Notebook). Since not all of the acquired fragment ion spectra were interpretable, the quality of the spectra was categorized based on the intensity of the most intense ARP reporter ion at *m/z* 227.0849, corresponding to the biotin moiety. The data association and subsequent processing was done in R and are described in the R Notebook provided with this publication. Here, the biotin fragment ion served as a proxy for the overall intensity of the relevant ions of the ARP-peptides, with the low intensity biotin fragment ion signals associated with noisy spectra. Finally, histograms of the fragment ion spectra identified by mgfHunter were, considering the type of peptide validation and the origin of the DDA method.

Re-analysis of data set reported under the ProteomeXchange identifier PXD002966 (Havelund et al[4])

The raw mass spectrometry files QHF0006.raw, QHF0009.raw, QHF0012.raw, QHF0013.raw, and QHF0415.raw for human plasma samples reported by Havelund *et al*.[4] were downloaded from the ProteomeXchange folder with the ID PXD002966 together with the corresponding

ProteomeDiscoverer search results (i.e., files with the .msf extension) with identifications provided by Mascot. Raw files were also searched with PEAKS PTM as described in the results section of this publication using the reduced long-chain biotin hydrazide modifications (lcBHZ) described by Havelund *et al*. Results were exported at 1% FDR at the peptide level and converted by PEAKS into .mzXML files. Both ProteomeDiscoverer and PEAKS Studio results were used to create DDA spectral libraries to generate precursor ion targets for analysis with Skyline. XICs were generated with a 20 ppm mass error for precursor ions and 5 min ID time shifts. Inspection of the spectral library generated from the Mascot results indicated that file QHF0015.raw used for identification was most likely uploaded incorrectly and therefore excluded from analysis. After refining the chromatographic peak picking, the coefficient of variation (CV) of the precursor signal areas was evaluated (Figure S14). Note that the peptide validation for this dataset was not as detailed as that used for the dataset presented in the main text.

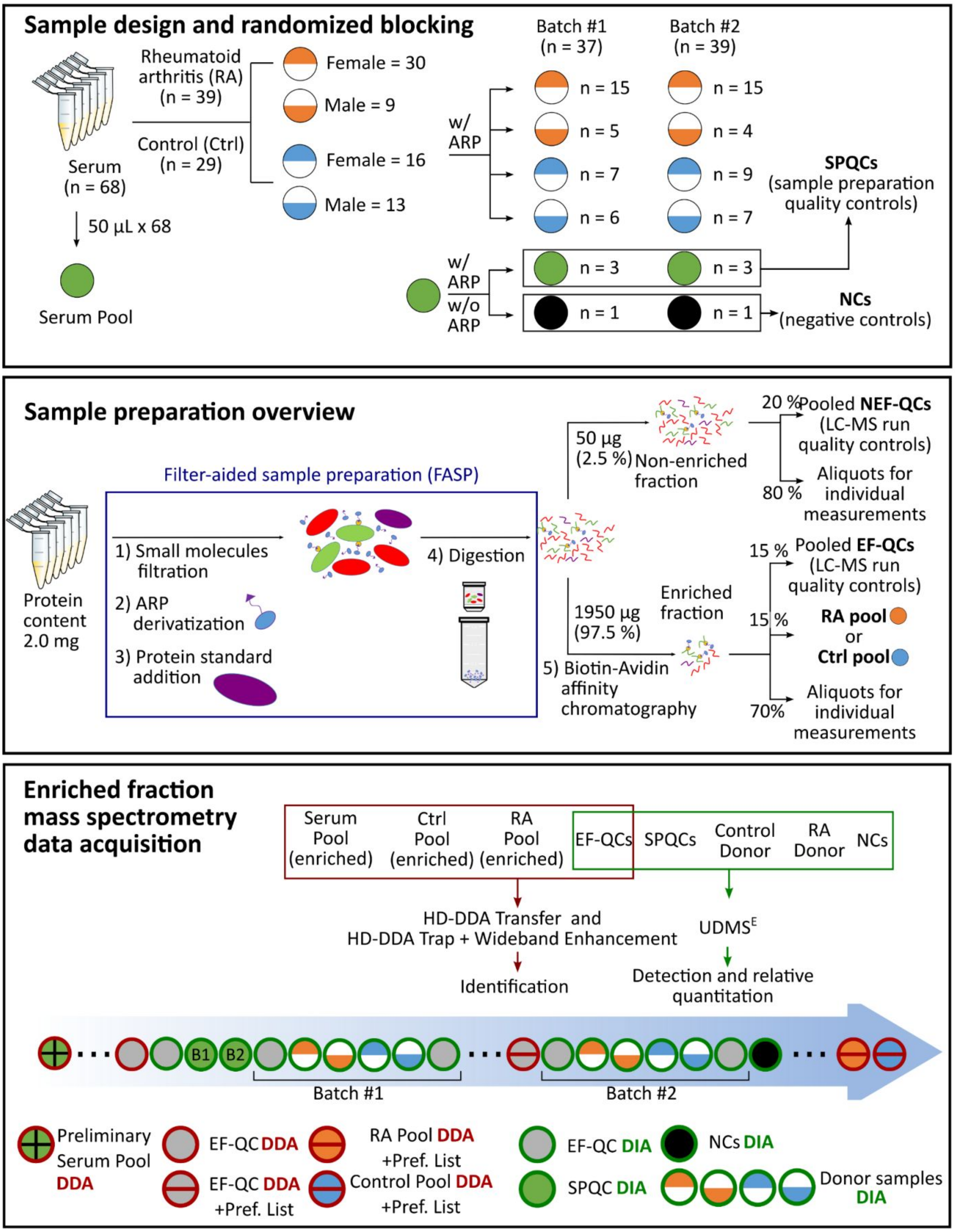

Figure S1. Experimental design of the present study. Sample design (top panel): Donor samples from 39 rheumatoid arthritis (RA) patients and 29 healthy individuals (control, Ctrl) were block randomized into two batches based on gender and disease classification. Equal aliquots of all serum samples were mixed (serum pool) to prepare three sample replicates for each batch (sample preparation quality controls, SPQCs). An additional aliquot was added to each batch, which was prepared in parallel with all other samples, but without the addition of ARP (negative control). Sample preparation using a modified FASP protocol (middle panel): Serum samples containing 2.0 mg of protein were derivatized with ARP, ultrafiltered (10 kDa cutoff), reduced, alkylated, and digested with trypsin. An aliquot (50 µg protein)

was taken for analysis and the remaining sample was enriched by avidin affinity chromatography. Aliquots from each fraction were combined as matrix-specific LC-MS run quality controls to monitor instrumental drift, i.e. enriched fraction quality controls (EF-QCs) and non-enriched fraction quality controls (NEF-QCs). Additional cohort-specific enriched digest pools, i.e., the Ctrl pool and the RA pool, were prepared from the enriched fractions. Data acquisition (bottom panel): EF-QCs and the enriched Ctrl and RA pools were analyzed by two data dependent acquisition (DDA) methods using precursor ion preference lists to generate DDA spectral libraries of ARP-peptides. Additionally, ARP-peptides identified in the ProteomeXchange data set deposited under the identifier PXD023738 were cross-referenced based on their indexed retention time (iRT). For quantitative interrogation, EF-QCs, NEF-QCs, SPQCs, NCs and individual RA and Ctrl samples were measured with $UDMS^E$, a wideband data independent acquisition (DIA). Initial DDA measurements were used to condition the system, followed by SPQCs from Batch #1 (B1) and Batch #2 (B2). Next, all donor samples from B1 and B2 were measured, interspersed with EF-QCs/NEF-QCs every 10 samples.

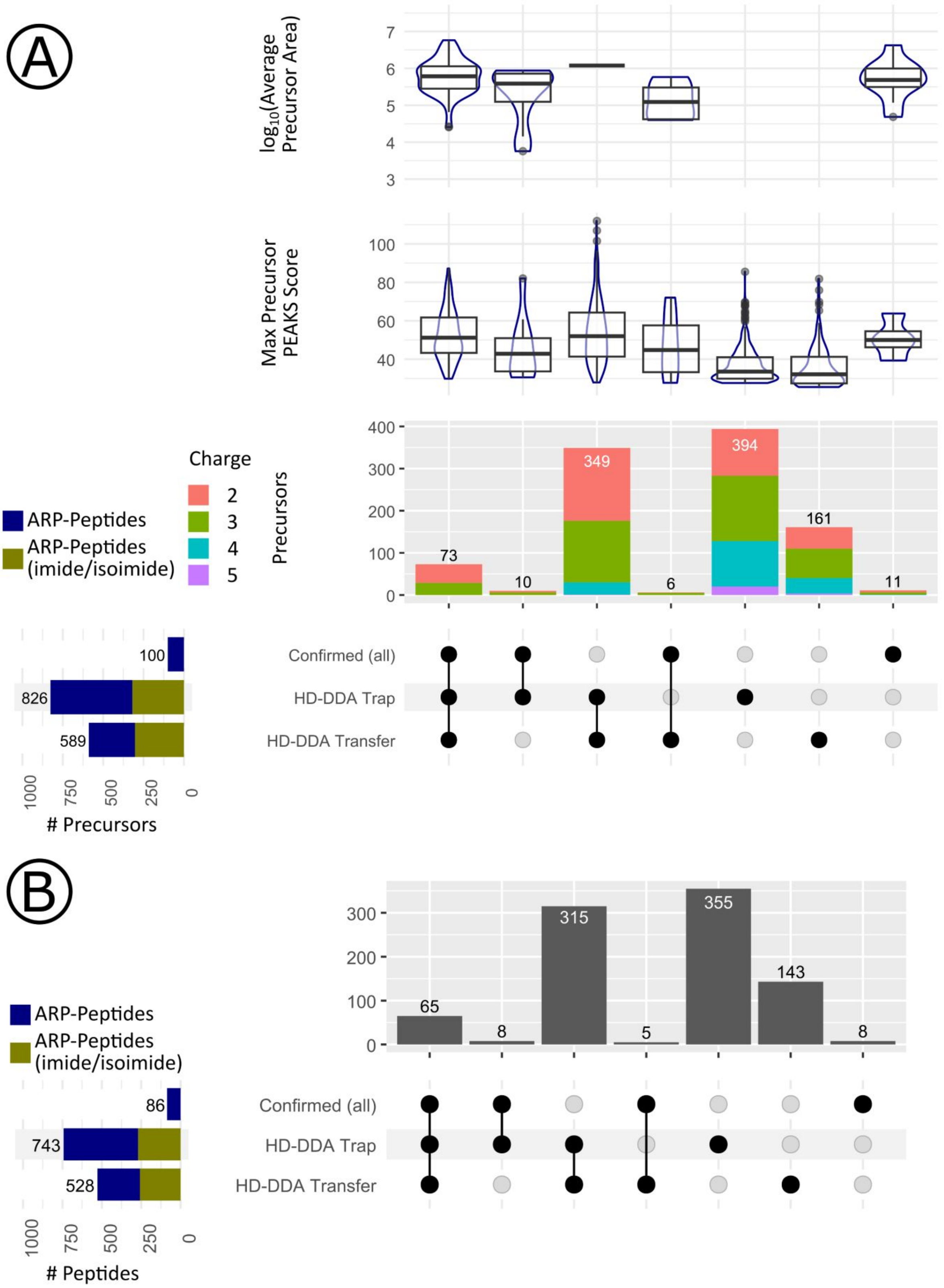

Figure S2. Overlap of identified precursor ions (A) and peptides (B) carrying ARP-derivatized modifications based on DDA results. Peptides and precursors not detected in the HD-DDA Trap and HD-DDA Transfer datasets acquired in this study correspond to ARP-peptides from a previous dataset with ProteomeXchange ID: PXD023738. This was confirmed in the UDMS^E data using peptide iRT and precursor and fragment ion isotope signal profiles. "Confirmed (all)" corresponds to the filtered carbonyl ARP-derivatized peptides identified in the Skyline document ARP-Serum_RA_enriched_filtered.sky available at https://panoramaweb.org/HumanSerumCarbonylationRA.url.

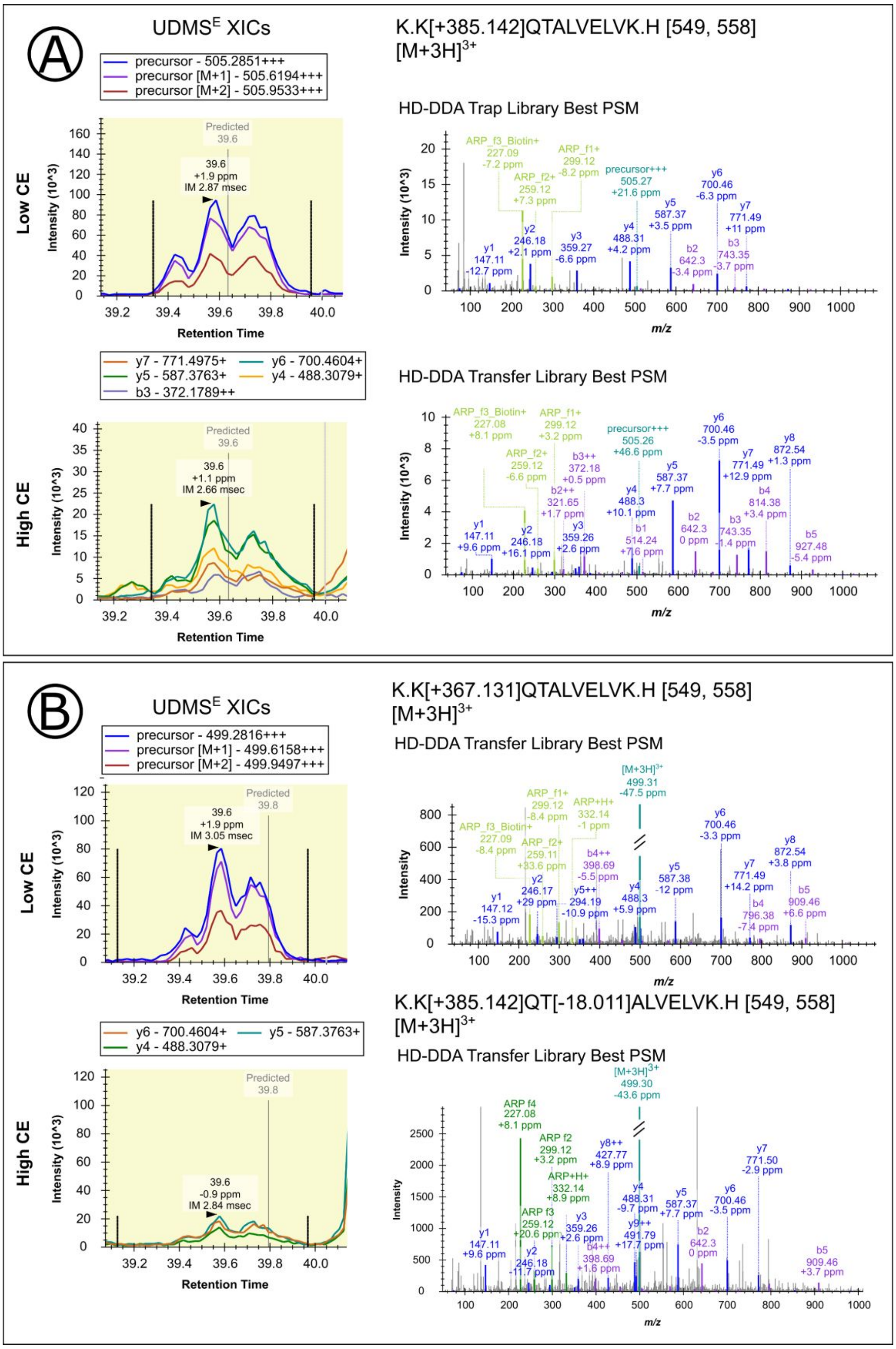

Figure S3. Precursor ion signal and fragment ion spectra of the triply protonated HSA peptide K[+385.142]QTALVELVK (A) and ambiguous peptide K[+385.142]QT[-18.011]ALVELVK or K[+367.131]QTALVELVK (B). Left panels show the zoomed XICs generated from UDMS^E data acquired by low and high collision energy scans (Low/High CE) for replicate S4_22_10_65.raw. Right panels show the corresponding peptide spectral matches from DDA spectral libraries. XICs of peptides K[+385.142]QT[-18.011]ALVELVK and K[+385.142]QTALVELVK had exact retention time overlap, indicating that the water loss of the former results from in-source fragmentation of peptide K[+385.142]QTALVELVK.

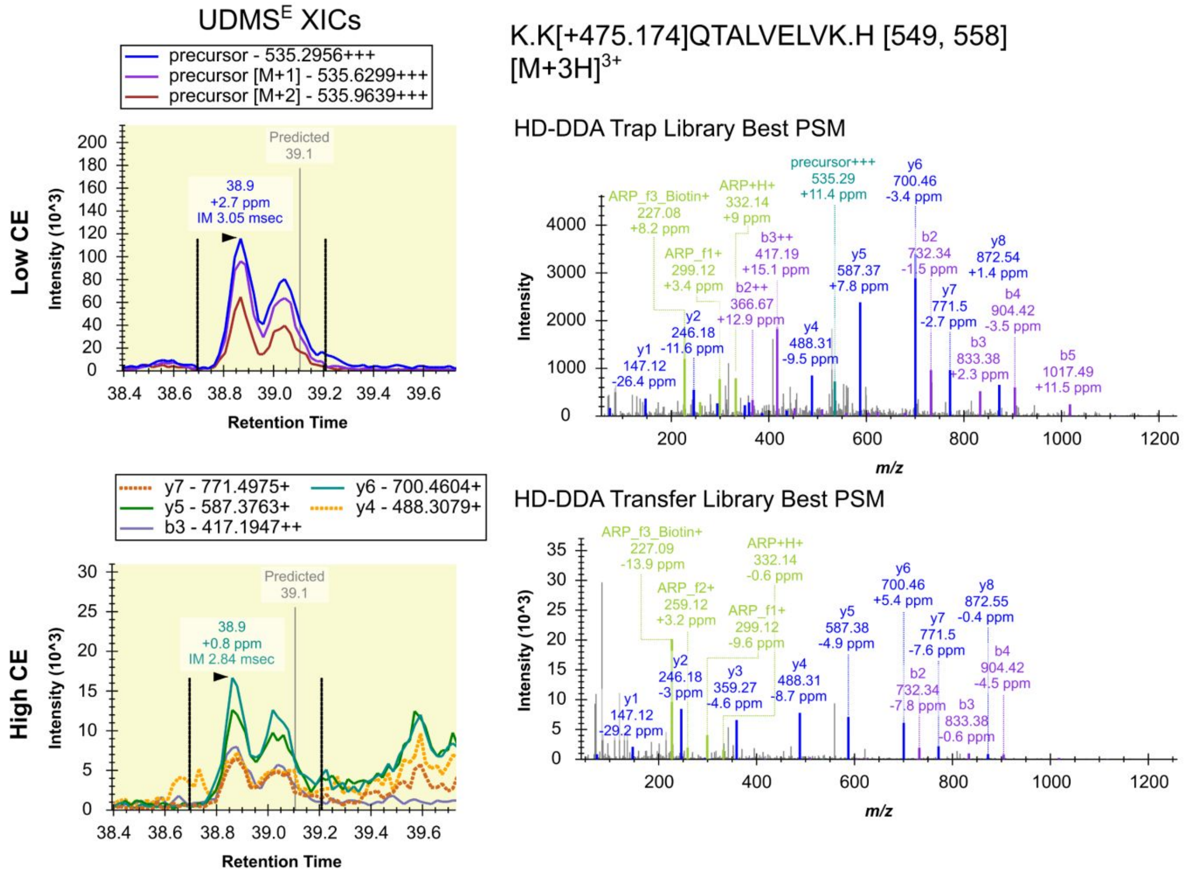

Figure S4. Precursor ion signal and fragment ion spectra of the triply protonated HSA peptide K[+475.174]QTALVELVK. Left panels show the zoomed XICs generated from UDMS^E data acquired by low and high collision energy scans (Low/High CE) for replicate S4_22_10_44.raw. Right panels show the corresponding best peptide spectral matches (PSMs) obtained from the spectral libraries generated with HD-DDA Trap and HD-DDA Transfer methods, respectively. Partially resolved XICs indicate the presence of different modified peptide isomers, probably corresponding to 1- and 3-deoxyglucosone adducts.

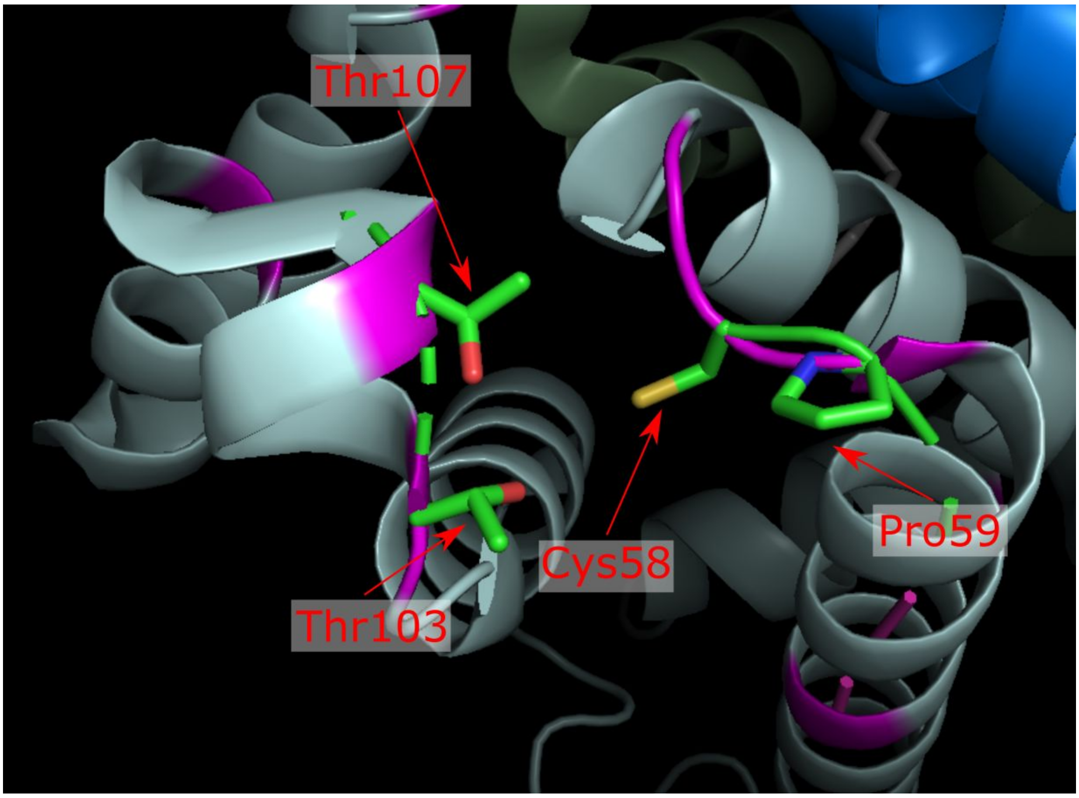

Figure S5. Carbonylation sites identified at Pro59, Thr103, and Thr107 near the metal ion binding site Cys58[5] are highlighted as a stick representation. The protein model shown corresponds to the protein data bank (PDB) identifier 1E7E. Visualization was performed with PyMOL v 2.5.4.

Observed precursor *m/z* = 671.8408
z = 2
Observed neutral mass = 1341.6660 Da

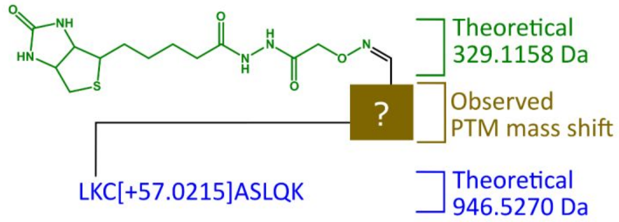

Observed PTM mass shift =
+ Observed neutral mass
- Theoretical unmodified peptide mass
- Theoretical ARP derivatization mass
+ Oxygen
Observed PTM mass shift = 82.02

Figure S6. Likely fragmentation scheme of the doubly protonated HSA peptide LK[+395.137]C[+57.021]ASLQK (residues 222-229).

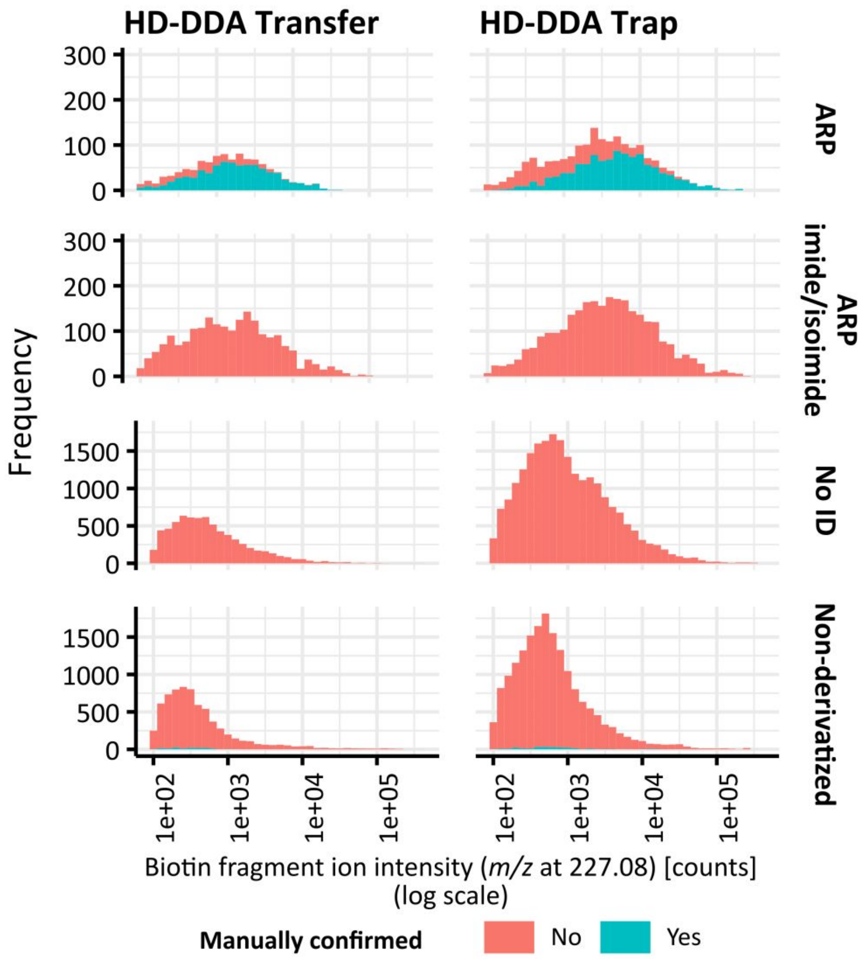

Figure S7. Histograms showing the signal intensities of ARP reporter ions. All fragment ion spectra selected by mgfHunter were classified with respect to the annotation provided by PEAKs and the type of peptide spectrum match identified, i.e., non-derivatized peptide sequences, ARP-peptide sequences, or no annotation. ARP-peptide sequences matched to proposed imide/isoimide modifications with mass shifts of +313.120862 and +314.104876 Da at Asp/Glu and Asn/Gln residues, respectively. ARP-peptide sequences containing these mass shifts were not manually confirmed and were not further considered in the current study.

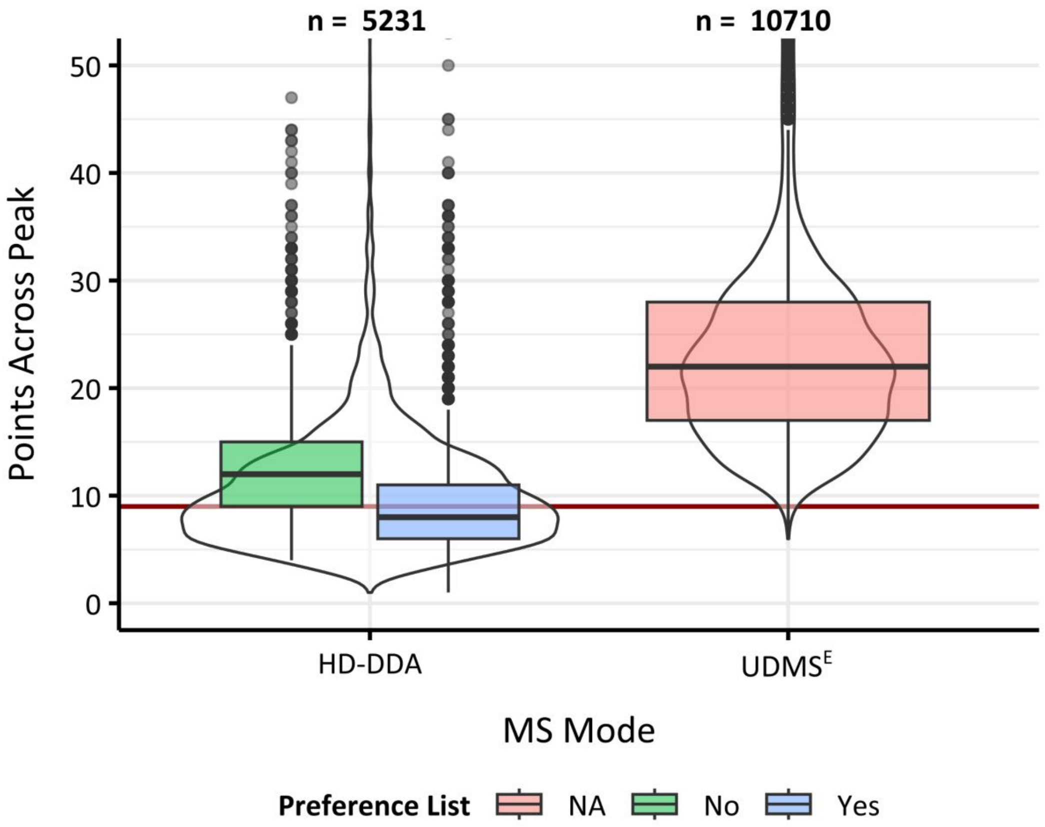

Figure S8. Distribution of the number of data points over the precursor signal in XICs. Boxplot boundaries represent the first and third quartiles and dark lines indicate the median. Violin plots show the area distribution of 5124 and 10583 signals integrated in HD-DDA and UDMS^E data, respectively. All data was extracted from filtered carbonyl ARP-derivatized peptides identified in the Skyline document ARP-Serum_RA_enriched_filtered.sky available at https://panoramaweb.org/HumanSerumCarbonylationRA.url.

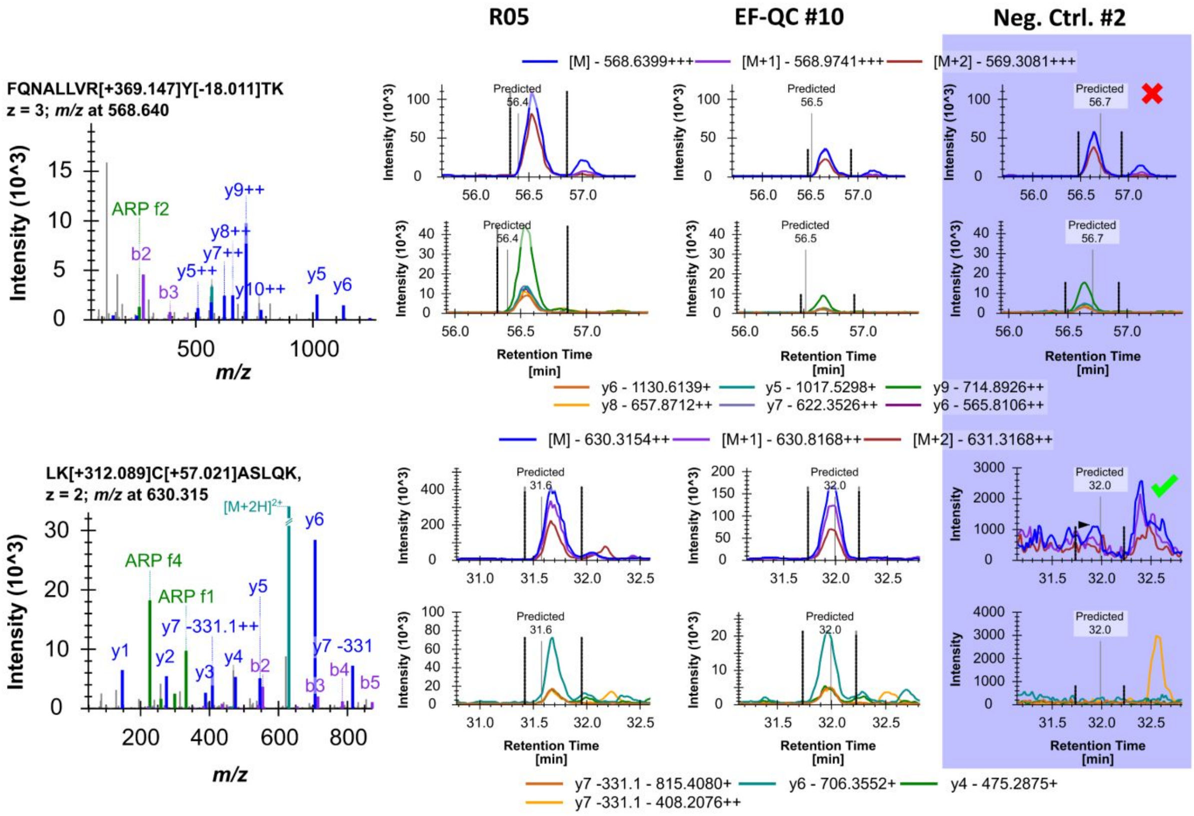

Figure S9. Examples of correctly and falsely identified ARP-labeled peptides. Left panels: The best HD-DDA PSMs of the peptides FQNALLVR[+369.147]Y[-18.011]TK and LK[+312.089]C[+57.021]ASLQK are shown. Both peptides passed the 1% FDR threshold at the peptide level. Right panels: XICs of the precursor (top) and fragment (bottom) are shown for each peptide detected in RA donor sample R05, enriched fraction QC (EF-QC) #10, and Negative Control #2 in Batch #2. The ARP-labeled peptide LK[+312.089]C[+57.021]ASLQK was detected in sample RO5 and EF-QC #10, but not in the negative control sample. In contrast, the signals indicative of ARP-labeled FQNALLVR[+369.147]Y[-18.011]TK in sample RO5 and EF-QC #10 were most likely due to other coeluting substances, as they were also detected in the Neg. Ctrl. Indeed, the PSM missed an intense biotin fragment at *m/z* 227.085 (ARP f1) and the y-series did not cover the modification site.

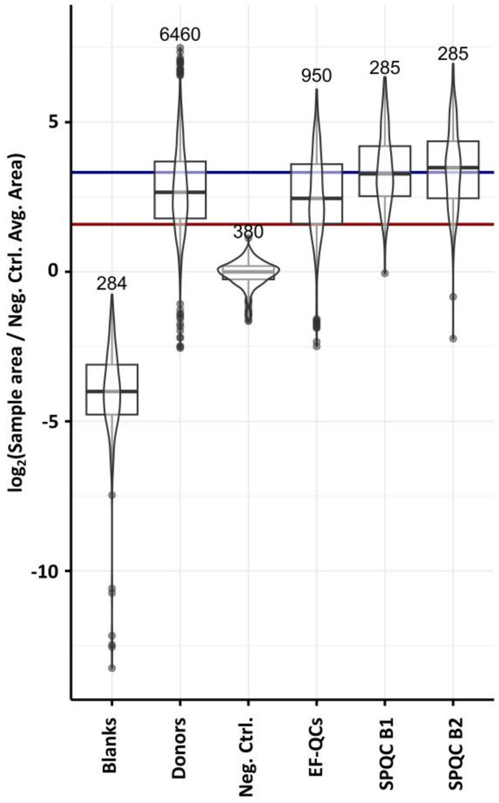

Figure S10. Fragment peak areas obtained by UDMS^E were calculated from the XICs of 86 ARP-derivatized peptides and normalized to the corresponding retention time aligned integration region in the underivatized negative control (Neg. Ctrl.). Box plots are grouped by sample type of normalized peak areas in all 68 donor and quality control serum samples. All data was extracted from filtered carbonyl ARP-derivatized peptides identified in the Skyline document ARP-Serum_RA_enriched_filtered.sky available at https://panoramaweb.org/HumanSerumCarbonylationRA.url.

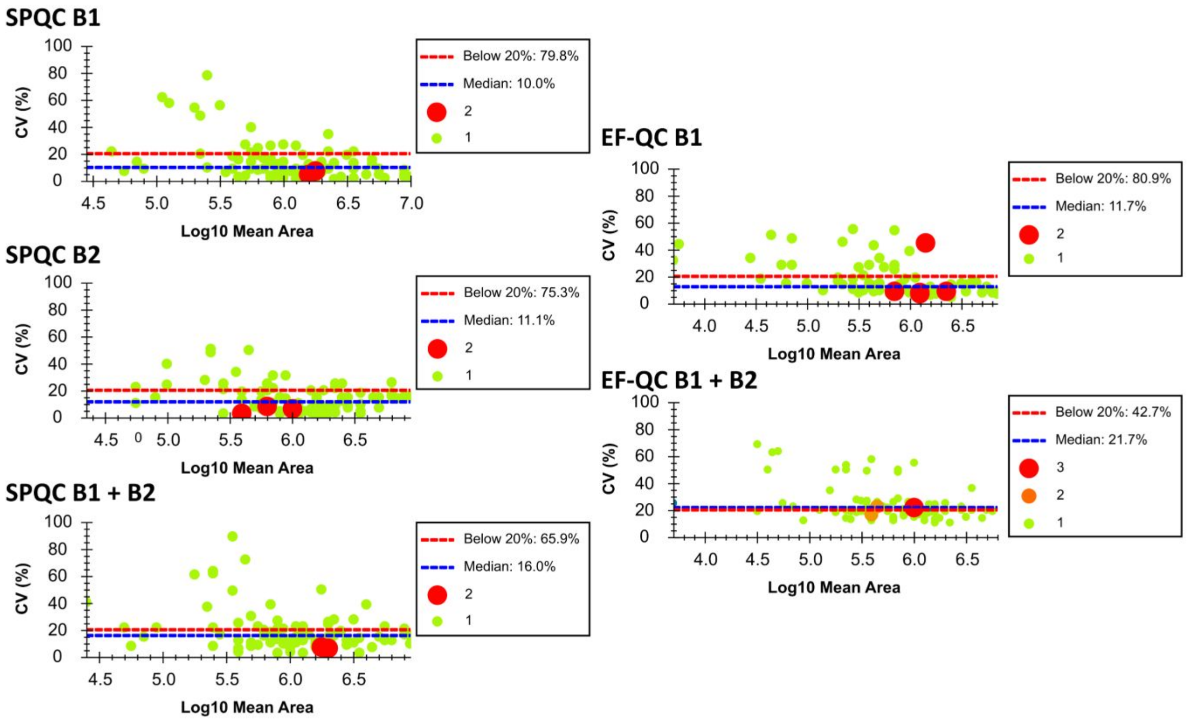

Figure S11. Histograms of the coefficient of variation (CV) of XIC peak areas of ARP-labeled peptides observed in SPQC and EF-QCs measured by UDMS^{E}. CVs were determined with different groupings considering the SPQCs of Batches #1 and #2, the combined results of both SPQCs, the enriched fraction QC samples in Batch 1 (EF-QC B1), and all EF-QCs of all donor samples (EF-QC B1 + B2). All data was extracted from filtered carbonyl ARP-derivatized peptides identified in the Skyline document "ARP-Serum_RA_enriched_filtered.sky" available at https://panoramaweb.org/HumanSerumCarbonylationRA.url.

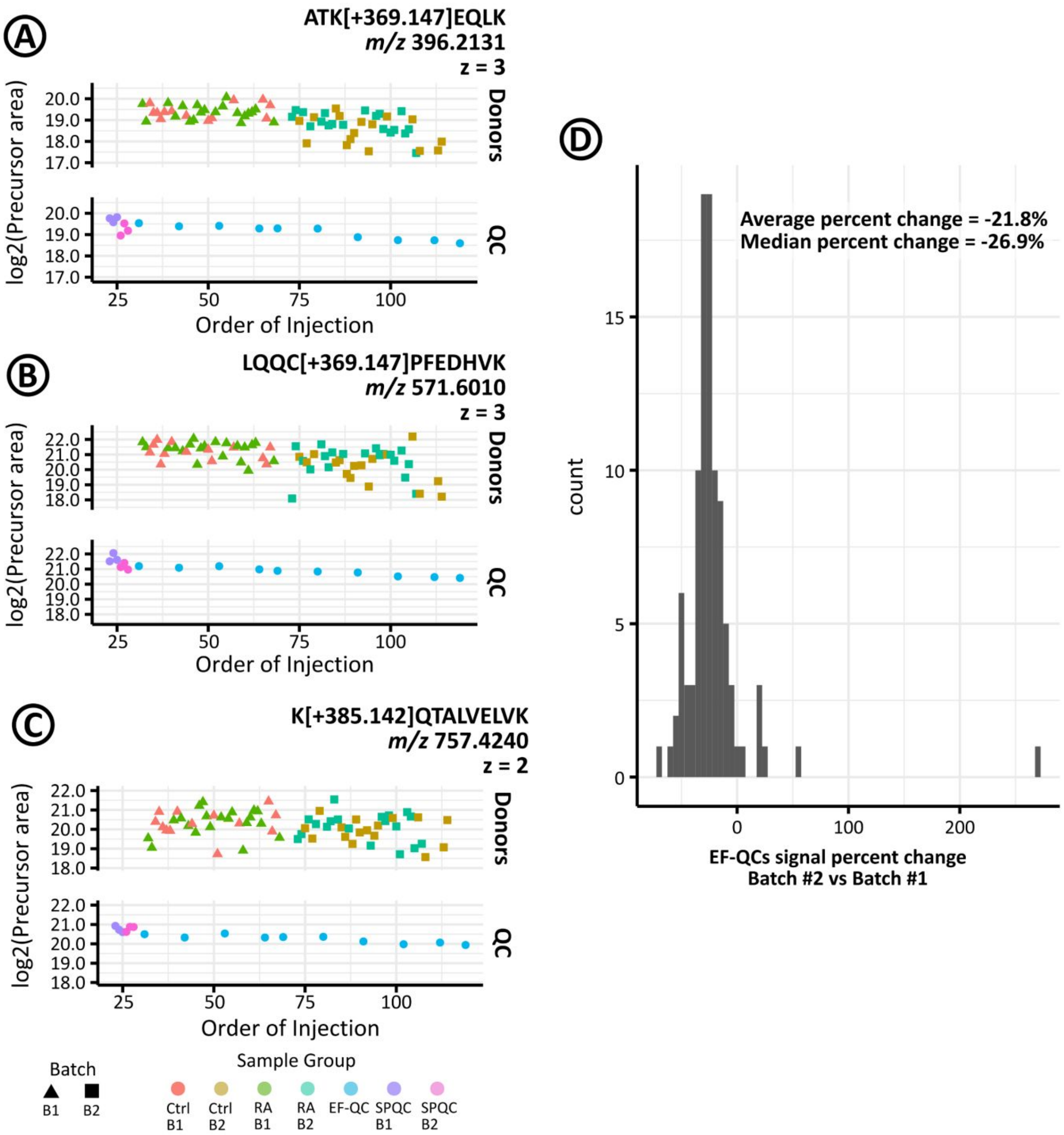

Figure S12. Signal distribution of ARP-peptides along the whole measurement time. Peak areas of precursor ion signals of ARP-labeled ATK[+369.147]EQLK (A), LQQC[+369.147]PFEDHVK (B), and K[+385.142]QTALVELVK (C). Sample groups correspond to Ctrl Batch #1 (Ctrl B1), Ctrl Batch #2 (Ctrl B2), RA Batch #1 (RA B1), RA Batch #2 (RA B2), enriched fraction QCs (EF-QCs), and sample preparation QCs of Batch #1 (SPQC B1) and batch #2 (SPQC B2). Average peak areas were determined for EF-QCs along batch #1 (B1) and batch #2 (B2). Signal change was determined for B2 against B1 EF-QCs and the signal change is presented as a histogram for all precursor ions evaluated (D); binwidth = 5. The following equation was used in the calculations for each precursor ion: Ratio = EFQC Batch #2 Average precursor area / EFQC Batch #2 Average precursor area. Percent change = (Ratio - 1) x 100. Average percent change = -21.8%. Median percent change = -26.1%. All data was extracted from filtered carbonyl ARP-derivatized peptides identified in the Skyline document "ARP-Serum_RA_enriched_filtered.sky" available at https://panoramaweb.org/HumanSerumCarbonylationRA.url.

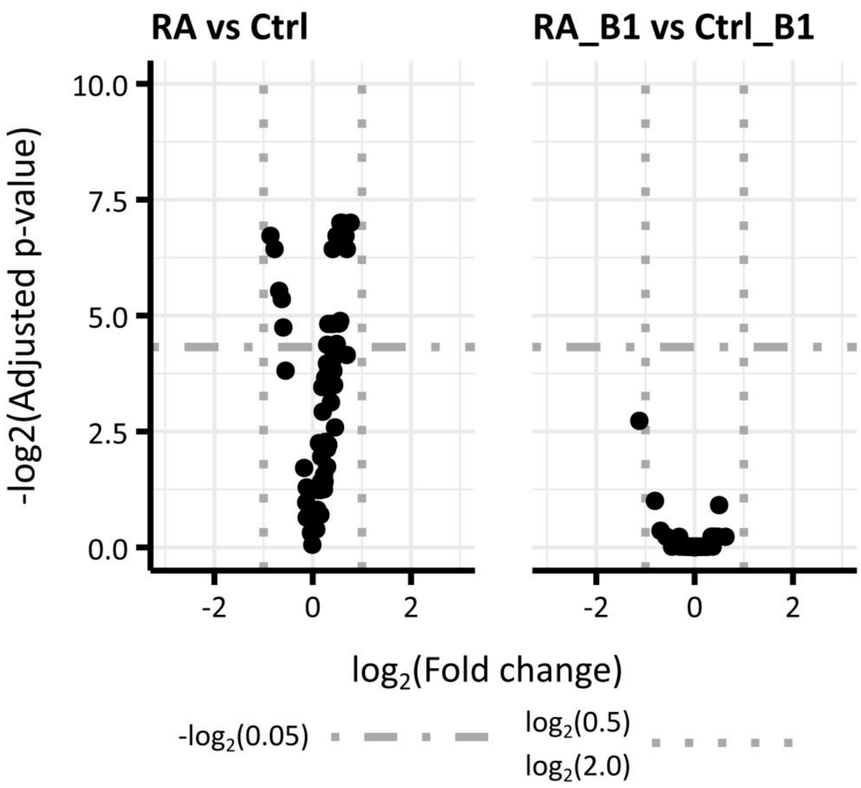

Figure S13. Volcano plots for differential analysis adjusted intensities of ARP-peptides using MStatsPTM. Adjusted p-values and fold changes are provided in Table S8. Statistical test summary considering batch effects between batch #1 and batch #2 (left volcano plot) and samples from batch #1 (right volcano plot). Data corresponding to filtered carbonyl ARP-derivatized peptides was extracted from the Skyline document “ARP-Serum_RA_enriched_filtered.sky” and data corresponding to non-derivatized peptides, measured prior to ARP-based enrichment, was extracted from the Skyline document “ARP-Serum_RA_NonEnriched_clinical_filtered.sky” available at https://panoramaweb.org/HumanSerumCarbonylationRA.url.

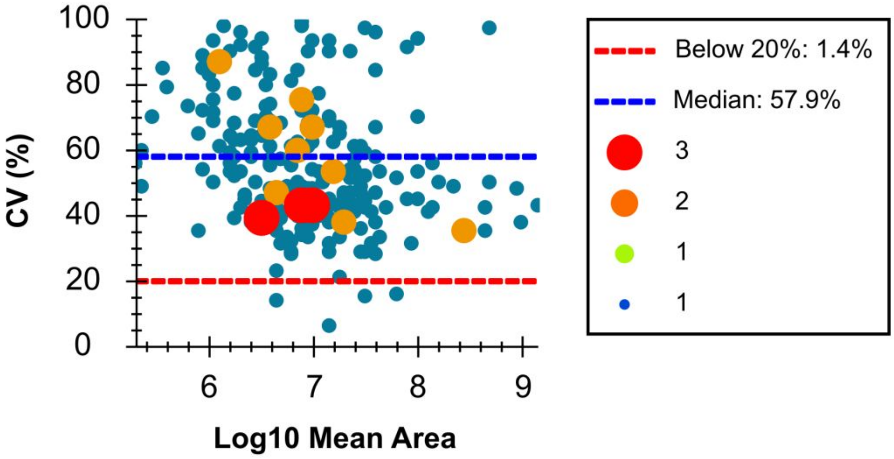

Figure S14. Coefficient of variation (CV) of the precursor area of peptides derivatized with long-chain biotin hydrazide (lcBHZ). Results correspond to the re-analysis of the data set deposited under the ProteomeXchange identifier PXD002966. The CV (%) is defined as the ratio of the precursor area standard deviation to the precursor area mean multiplied by 100. All data was extracted from filtered carbonyl lc-BHZ-derivatized peptides identified in the Skyline document "PXD002966_lc-BHZ_Plasma.sky" available at https://panoramaweb.org/HumanSerumCarbonylationRA.url.

References

(1) Helm, D.; Vissers, J. P. C.; Hughes, C. J.; Hahne, H.; Ruprecht, B.; Pachl, F.; Grzyb, A.; Richardson, K.; Wildgoose, J.; Maier, S. K.; Marx, H.; Wilhelm, M.; Becher, I.; Lemeer, S.; Bantscheff, M.; Langridge, J. I.; Kuster, B. Ion Mobility Tandem Mass Spectrometry Enhances Performance of Bottom-up Proteomics. *Molecular & Cellular Proteomics* **2014**, *13* (12), 3709–3715. https://doi.org/10.1074/mcp.M114.041038.

(2) Rojas Echeverri, J. C.; Milkovska-Stamenova, S.; Hoffmann, R. A Workflow towards the Reproducible Identification and Quantitation of Protein Carbonylation Sites in Human Plasma. *Antioxidants* **2021**, *10* (3), 369. https://doi.org/10.3390/antiox10030369.

(3) Rojas Echeverri, J. C.; Volke, D.; Milkovska-Stamenova, S.; Hoffmann, R. Evaluating Peptide Fragment Ion Detection Using Traveling Wave Ion Mobility Spectrometry with Signal-Enhanced MS E (SEMS^E). *Anal Chem* **2022**, *94* (31), 10930–10941. https://doi.org/10.1021/acs.analchem.2c00461.

(4) Havelund, J. F.; Wojdyla, K.; Davies, M. J.; Jensen, O. N.; Møller, I. M.; Rogowska-Wrzesinska, A. A Biotin Enrichment Strategy Identifies Novel Carbonylated Amino Acids in Proteins from Human Plasma. *J Proteomics* **2017**, *156*, 40–51. https://doi.org/10.1016/j.jprot.2016.12.019.

(5) Bal, W.; Sokołowska, M.; Kurowska, E.; Faller, P. Binding of Transition Metal Ions to Albumin: Sites, Affinities and Rates. *Biochim Biophys Acta Gen Subj* **2013**, *1830* (12), 5444–5455. https://doi.org/10.1016/j.bbagen.2013.06.018.
